# Transcriptome profiles revealed molecular mechanisms of alternating temperatures in breaking the epicotyl morphophysiological dormancy of *Polygonatum sibiricum* seeds

**DOI:** 10.1186/s12870-021-03147-7

**Published:** 2021-08-12

**Authors:** Dengqun Liao, Ruipeng An, Jianhe Wei, Dongmei Wang, Xianen Li, Jianjun Qi

**Affiliations:** 1 Institute of Medicinal Plant Development, Chinese Academy of Medical Sciences & Peking Union Medical College, Beijing, 100193 China; 2 College of Life Science, Hebei Agricultural University, Baoding, 071000 Hebei China; 3 The Key Laboratory of Plant Physiology and Molecular Pathology, Hebei province, Hebei Agricultural University, Baoding, 071000 Hebei China

**Keywords:** *Polygonatum sibiricum* red, Epicotyl morphophysiological dormancy, Temperature stratification, SMRT (single-molecule real-time) sequencing, Full-length transcriptome, RNA sequencing, Gene expression, Hormone, Transcription factor, Seed germination-related gene

## Abstract

**Background:**

To adapt seasonal climate changes under natural environments, *Polygonatum sibiricum* seeds have a long period of epicotyl morphophysiological dormancy, which limits their wide-utilization in the large-scale plant progeny propagation. It has been proven that the controlled consecutive warm and cold temperature treatments can effectively break and shorten this seed dormancy status to promote its successful underdeveloped embryo growth, radicle emergence and shoot emergence. To uncover the molecular basis of seed dormancy release and seedling establishment, a SMRT full-length sequencing analysis and an Illumina sequencing-based comparison of *P. sibiricum* seed transcriptomes were combined to investigate transcriptional changes during warm and cold stratifications.

**Results:**

A total of 87,251 unigenes, including 46,255 complete sequences, were obtained and 77,148 unigenes (88.42%) were annotated. Gene expression analyses at four stratification stages identified a total of 27,059 DEGs in six pairwise comparisons and revealed that more differentially expressed genes were altered at the Corm stage than at the other stages, especially Str_S and Eme. The expression of 475 hormone metabolism genes and 510 hormone signaling genes was modulated during *P. sibiricum* seed dormancy release and seedling emergence. One thousand eighteen transcription factors and five hundred nineteen transcription regulators were detected differentially expressed during stratification and germination especially at Corm and Str_S stages. Of 1246 seed dormancy/germination known DEGs, 378, 790, and 199 DEGs were associated with *P. sibiricum* MD release (Corm vs Seed), epicotyl dormancy release (Str_S vs Corm), and the seedling establishment after the MPD release (Eme vs Str_S).

**Conclusions:**

A comparison with dormancy- and germination-related genes in *Arabidopsis thaliana* seeds revealed that genes related to multiple plant hormones, chromatin modifiers and remodelers, DNA methylation, mRNA degradation, endosperm weakening, and cell wall structures coordinately mediate *P. sibiricum* seed germination, epicotyl dormancy release, and seedling establishment. These results provided the first insights into molecular regulation of *P. sibiricum* seed epicotyl morphophysiological dormancy release and seedling emergence. They may form the foundation of future studies regarding gene interaction and the specific roles of individual tissues (endosperm, newly-formed corm) in *P. sibiricum* bulk seed dormancy.

**Supplementary Information:**

The online version contains supplementary material available at 10.1186/s12870-021-03147-7.

## Background

*Polygonatum sibiricum* Red (Huangjing in Chinese) is an edible perennial lily species with medicinal properties. The rhizome of this plant together with those of *P. kingianum* Col1. et Hemsl and *P. cyrtonema* Hua have been used as a traditional Chinese medicine for nourishing *Qi* and *Yin* and for enhancing spleen, lung, and kidney functions for approximately 1600 years [1–4]. Modern pharmacological studies have proved that polygonati rhizoma can improve immunity as well as lower blood sugar and lipid levels. Additionally, its anti-viral and anti-tumorigenic properties have been confirmed. Thus, polygonati rhizoma may be useful for developing novel drugs and health products relevant for treating age-related diseases, hypolipidemia, atherosclerosis, osteoporosis, liver diseases, diabetes mellitus, lung diseases, coughs, fatigue, and insomnia [3–8]. *P. sibiricum* is one of three *Polygonatum* species used as a source of polygonati rhizoma listed in the Pharmacopoeia of the People’s Republic of China. As an edible and medicinal plant, the market demand for polygonati rhizoma has increased substantially, with more than 4000 tons produced annually. For many years, most of the polygonati rhizoma on the market was derived from wild resources, which has depleted the limited wild resources due to the long growth period to produce harvestable rhizomes (3–4 years for rhizome propagation and 5–6 years from seed propagation). To satisfy the increasing market demand and ensure the sustainable production and supply of polygonati rhizoma and to protect and preserve wild resources, *Polygonatum* plants are now widely cultivated in China. *P. cyrtonema* Hua is mainly cultivated in the Yangtze River basin and in the southern region, whereas *P. kingianum* Col1. et Hemsl and *P. sibiricum* Red are primarily cultivated in Yunnan province and northern China, respectively.

**Fig. 1:**
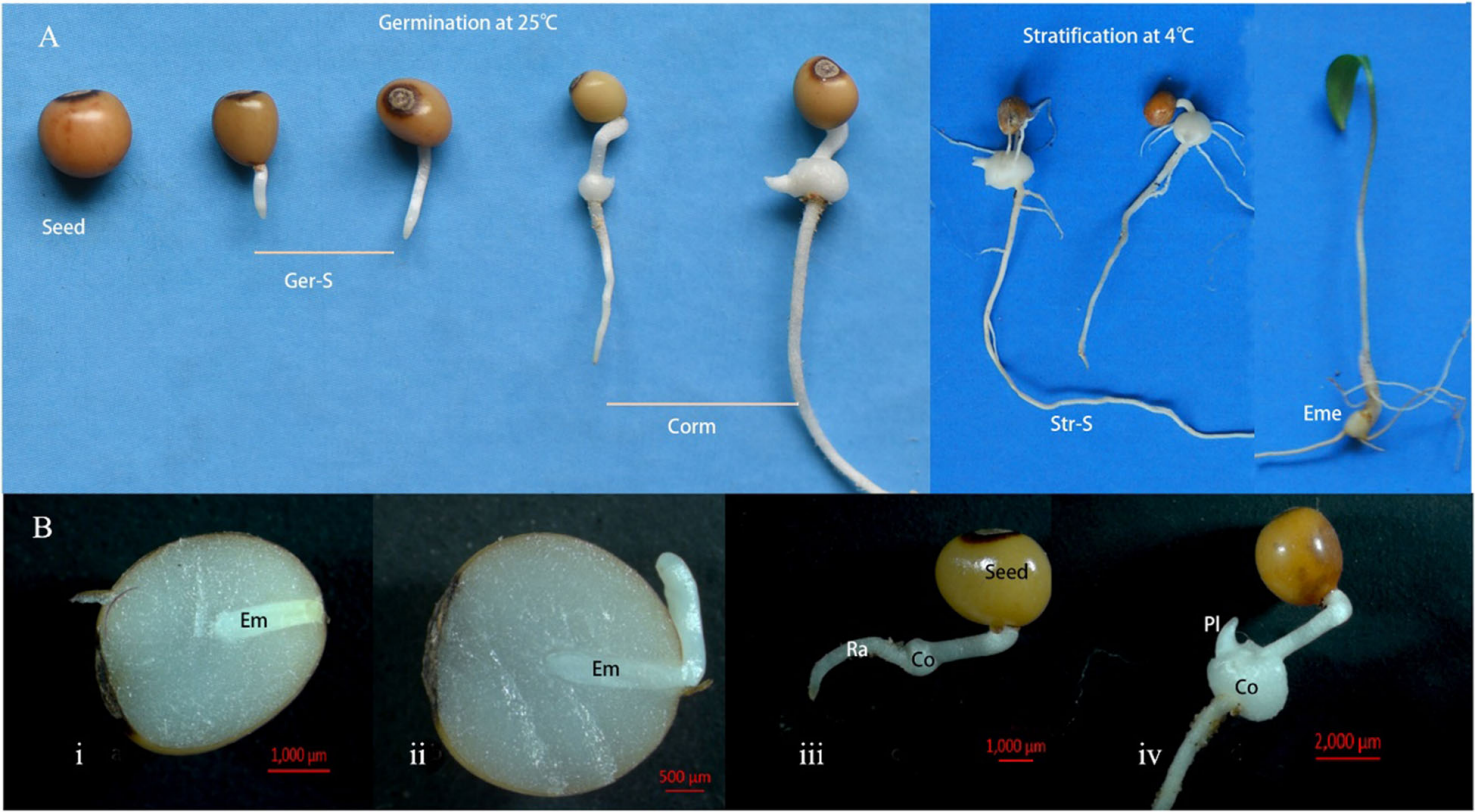
Temperature stratification treatments promote *P. sibiricum* seed germination and seedling establishment (**A**) and embryo development (**B**). **A** five main overall stages of *P. sibiricum* seeds during stratification: dormant mature seed before a warm stratification (Seed); seed germination with the embryo extruding from a hilum (Ger-S) and cormlet formation (Corm) at 25 °C stratification; the radicle and lateral roots form and the corm continues to grow at 4 °C stratification (Str_S); and seedling emergence with a leaf after transferring to 25 °C (Eme). B, four major stages of embryo development during the germination at 25 °C: i) undeveloped club-shaped embryo in a dormant seed at maturation; ii) immature embryo elongates and is extruded from the hilum, indicating the dormant *P. sibiricum* seed has germinated; iii) hypocotyl increases in size and a cormlet forms on the radicle; and iv) corm continues to grow and differentiate, which is accompanied by the emergence of a plumule. Em: embryo; Ra: radicle; Co: corm; Pl: plumule

*P. sibiricum* can be propagated from its rhizomes or seeds. Because of the low efficiency of rhizome propagation and the considerable time needed for rhizome growth (3–4 years), seed propagation may be the superior option for the large-scale cultivation of *P. sibiricum* plants. However, *P. sibiricum* seeds at maturation have morphophysiological dormancy (MPD) and require a long dormancy period of about 15 months under natural conditions to complete the morphological and physiological dormancy-related processes before seedling emergence. It was found that seed structure, endogenous inhibitors, and underdeveloped embryo at maturation influenced *P. sibiricum* seed dormancy and germination [9]. Methods for effectively stimulating the germination of *P. sibiricum* dormant seeds, including soaking, exogenous hormone treatments, and temperature stratifications, were also evaluated. It was observed that *P. sibiricum* seeds exposed to 25 °C could germinate in 30–60 days and form corm tissue, after which a low-temperature (4 °C) treatment is needed to break the epicotyl dormancy in about 60 days before seedling emergence [10–12]. Similar to germination process of MPD seeds of *Lilium dahuricum* [13], *Lilium polyphyllum* [14], and *Arisaema dracontium* especially in cold regions [15, 16], *P. sibiricum* seeds underwent corm formation and plumule development, and required nutrients transported from endosperm reserves into the new corm tissue and a cold stratification prior to seedling establishment [12, 17]. This type of seed dormancy and germination is quite different from the corresponding processes of many other MPD seeds such as *Paris polyphylla* [18], *Panax quinquefolius* [19], and *Paeonia suffruticosa* Andr [20]. For *Paris polyphylla*, *Panax quinquefolius* and *Paeonia suffruticosa* dormant seeds, their embryo differentiation into a visible radicle, plantule, hypocotyl or epicotyl, and/or cotyledons occurs inside the seed before germination. In contrast, the immature club-shaped embryo in *P. sibiricum* seeds elongates under suitable warm and moist conditions and pushes the radicle, hypocotyl, and plantule primordium out of the endosperm through the hilum; the visible plumule then differentiates and develops on the protuberant hypocotyl (defined as the “corm”) and stops growing until a cold stratification is exerted to release epicotyl dormancy [12, 17] (Fig. 1). Currently, the molecular basis of *P. sibiricum* seed dormancy release and germination, especially in terms of corm formation and epicotyl dormancy, remains unclear. Because of a lack of genome and transcriptome sequence information, in this study, we applied a single-molecule real-time sequencing (SMRT-seq) strategy to analyze a pooled total RNA sample derived from six stages to generate a complete and full-length *P. sibiricum* transcriptome during seed germination and seedling emergence. The transcript isoforms served as the reference sequences for the functional annotation of *P. sibiricum* genes and in the subsequent comparative transcriptome study. Additionally, we conducted an Illumina short-read sequencing-based comparison of the transcriptomic profiles in four key stages of *P. sibiricum* seeds during dormancy release. The potential genes related to the MPD release of *P. sibiricum* mature seeds following a warm stratification to develop the corm and the subsequent epicotyl dormancy release by a cold stratification were separately identified. These results provided the first insights into the molecular regulation of *P. sibiricum* seed MPD release, germination, and seedling emergence.

## Results

### SMRT-seq analysis of *P. sibiricum* transcriptome during seed dormancy, germination, and seedling emergence

**Fig. 2:**
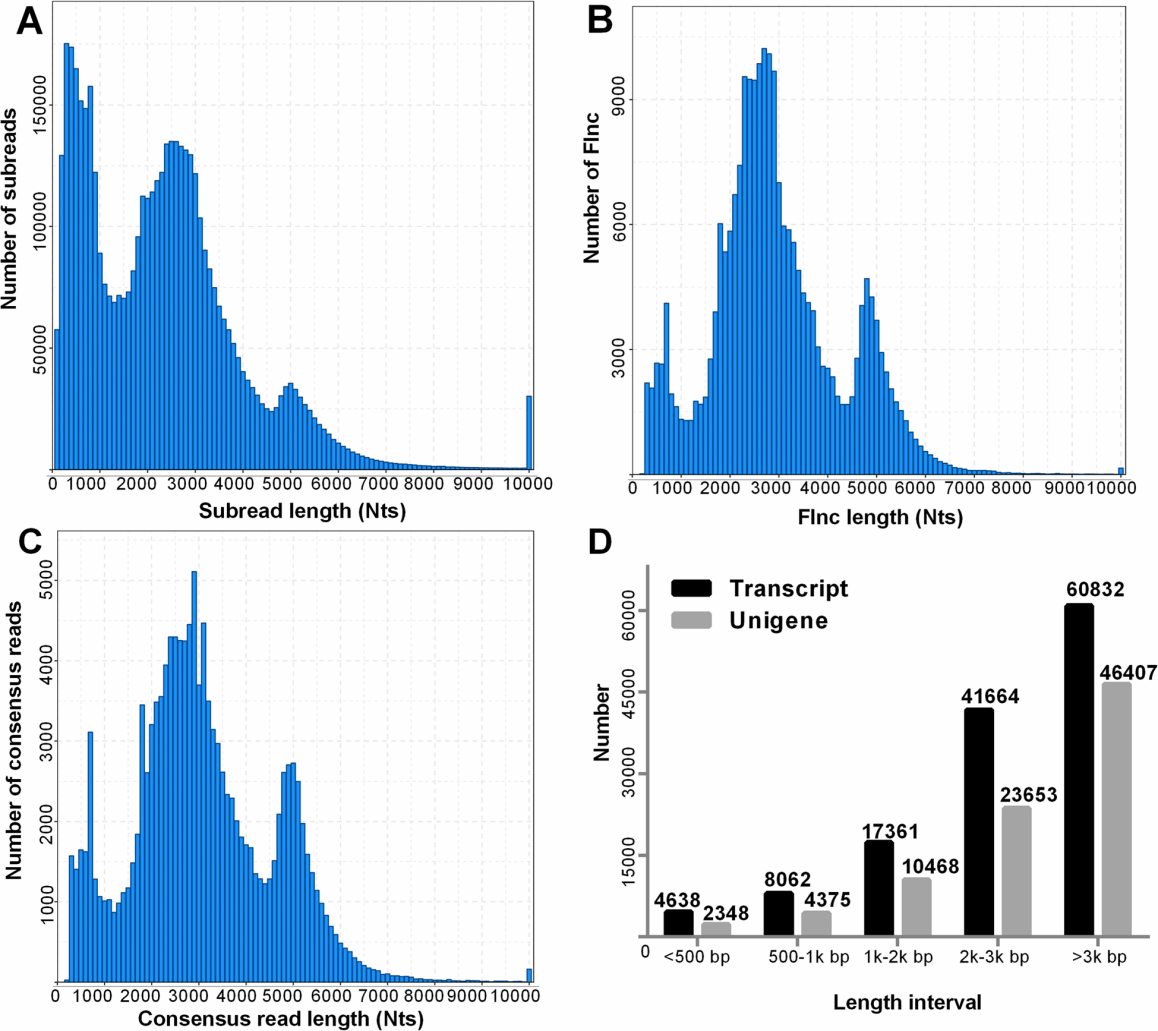
Number and length distribution of PacBio SMRT sequencing data for *P. sibiricum* transcriptome. **A** Number and length distribution of subreads; **B** Number and length distribution of Flnc reads; **C** Number and length distribution of consensus reads (transcripts); **D** Distribution of the 132,557 transcripts and 82,571 unigenes at different length intervals

To obtain a sequenced *P. sibiricum* transcriptome during seed dormancy, germination, and seedling emergence, the PacBio RSII platform was used to perform a SMRT-seq analysis of a pooled RNA sample from six different seed developmental stages [dormant mature seed, early germinating seed during a warm stratification (Ger-S), germinated seed with a corm during a warm stratification (Corm), early stage (about 4 weeks) of a cold stratification (Str), late stage (about 8 weeks) of a cold stratification (Str_S), and seedling emergence during a warm stratification (Eme)]. A total of 4,789,895 subreads (11.36 Gb) were generated, with an average length of 2373 bp and an N50 of 3166 bp (Table 1). A total of 292,791 circular consensus sequences (CCS) were obtained using the SMRTlink 5.1 software and further classified into 49,909 non-full length reads and 239,376 full-length reads, of which 230,162 were full-length non-chimeric (Flnc) reads. An isoform-level clustering analysis yielded 132,557 consensus reads, with an average length of 3076 bp, an N50 of 3633 bp, and an N90 of 2098 bp after correcting errors using RNA sequencing (RNA-seq) data. Redundant sequences among 132,557 consensus reads were eliminated using the CD-HIT software, ultimately resulting in 87,251 unigenes. Approximately 82.45% of all unigenes (71,936) had only one transcript and 9.39% unigenes had two transcripts (Table S1). The length distributions of the subreads, Flnc reads, and consensus sequences are presented in Fig. 2A-C. Additionally, 46,407 unigenes (53.19% of the total) were longer than 3 kbp (Fig. 2D). Using the ANGLE pipeline, 84,177 unigenes were predicted as protein-coding sequences, of which 46,255 were identified as full-length sequences (i.e., a complete coding sequence as well as 5′ and 3′ untranslated regions). The length distribution of the predicted protein-coding sequences is provided in Figure S1.

**Table 1:** Summary of the *P. sibiricum* transcriptome SMRT sequencing data

Total Subread base (Gb)	11.36
Subread number	4,789,895
Average subread length (bp)	2373
N50 length (bp)	3166
Number of circular consensus sequence	292,791
Number of 5′ primer reads	267,809
Number of 3′ primer reads	270,084
Number of poly-A reads	265,089
Number of non-full-length reads	49,909
Number of full-length reads	239,376
Number of full-length non-chimeric reads	230,162
Average length of Flnc reads (bp)	2920
**Number of consensus reads**	**132,557**
Total nucleotides (bp) before and after correction	404,700,846/40769,903
Mean length before and after correction (bp)	3054/3076
Minimum length before and after correction (bp)	161/162
Maximum length before and after correction (bp)	14,162/14371
N50 length before and after correction (bp)	3598/3633
N90 length before and after correction (bp)	2059/2098
Number of unigenes	87,251

Note: 5′ primer reads refers to the reads with the 5′ primer; 3′ primer reads refers to the reads with the 3′ primer; poly-A reads refers to the reads with poly-A; full-length non-chimeric (Flnc) reads refers to non-chimeric reads with the 5′ primer, 3′ primer, and poly-A

### Functional annotation of *P. sibiricum* transcriptome

A total of 87,251 full-length unigenes were functionally annotated based on BLAST searches of the NCBI non-redundant protein (Nr), NCBI non-redundant nucleotide (Nt), Swiss-Prot, Protein family (Pfam), Clusters of Orthologous Groups (KOG/COG) of proteins, Gene Ontology (GO), and Kyoto Encyclopedia of Genes and Genomes (KEGG) databases. Using an E-cutoff value ≤1e-5 and the top hit for the searches, a total of 77,148 unigenes (88.42%) had significant sequence matches in at least one of the databases (Table S2). Among them, 25,521 unigenes (29.25%) matched sequences in all seven databases. The number and percentage of annotated *P. sibiricum* unigenes for the seven databases were as follows: Nr: 74,921 (85.87%), Nt: 54,583 (62.56%), Swiss-Prot: 61,678 (70.69%), Pfam: 41,807 (47.92%), KOG: 50,906 (58.34%), GO: 41,807 (47.92%), and KEGG: 73,525 (84.27%). Among *P. sibiricum* unigenes annotated using the Nr database, 62,681 and 11,610 were assigned to 182 monocot species and 175 dicot species, respectively (Table S3). Among 402 Viridiplantae species with sequence matches, the three species with the most matches to *P. sibiricum* unigenes were *Asparagus officinalis* (39,017, 52.13%), *Elaeis guineensis* (7561, 10.10%), and *Phoenix dactylifera* (6226, 8.31%), all of which are monocots. There are currently only 543 genes and 1732 protein sequences of *Polygonatum* species available in the NCBI databases. Our SMRT-seq analysis resulted in 544 *P. sibiricum* unigenes assigned to the Nr sequences of the following eight *Polygonatum* species: *P. biflorum* (1), *P. cyrtonema* (175), *P. involucratum* (1), *P. multiflorum* (143), *P. pubescens* (7), *P. roseum* (64), *P. sibiricum* (73), and *P. verticillatum* (79) (Table S4)*.* These 544 unigenes are 34.7–100% homologous to known sequences from *Polygonatum* species.

The KOG database was built with orthologous proteins encoded in the *Arabidopsis thaliana* (Arabidopsis) genome and the genomes of six other non-Viridiplantae species (http://www.ncbi.nlm.nih.gov/COG/). The BLASTx search of the KOG database matched 48,662 *P. sibiricum* unigenes with 7587 Arabidopsis orthologous proteins as well as 2244 *P. sibiricum* unigenes with 1279 orthologous proteins from non-Viridiplantae species. These 50,906 *P. sibiricum* unigenes were classified into 3209 orthologous protein clusters, and their distribution in 25 categories is presented in Figure S2. The largest KOG category was “general functional prediction only” (13,408, 15.37%), followed by “post-translational modification, protein turnover, chaperones” (5295, 6.07%) and “signal transduction mechanisms” (4766, 5.46%). The GO functional annotation resulted in 41,807 unigenes classified into 54 subcategories (Table S5) of the three main functional categories (Figure S3). Specifically, 26,629 unigenes were assigned to 25 biological processes, with “metabolic process,” “cellular process,” and “single-organism process” revealed as the largest subcategories.

Among 73,525 *P. sibiricum* unigenes annotated using the KEGG database, 34,513 were assigned KO identifiers, of which 22,381 unigenes were further mapped to 341 KEGG pathways (third level) (Table S6). A total of 9323 unigenes were assigned to the metabolic pathways, including “carbohydrate metabolism” (3240), “energy metabolism” (1969), “lipid metabolism” (1506), and “amino acid metabolism” (1934). Additionally, 4225 unigenes were assigned to cellular processes, of which 2871 were associated with “transport and catabolism” and 1238 were related to “cell growth and death.” Among the environmental information processing modules, 3862 unigenes were predicted to affect signal transduction pathways, including the “plant hormone signal transduction pathway” (699) and “MAPK signaling pathway—plant” (656), suggesting they may be useful for studying the regulatory effects of hormones on *P. sibiricum* seed germination.

### Differential expression of *P. sibiricum* genes during seed stratification

**Fig. 3:**
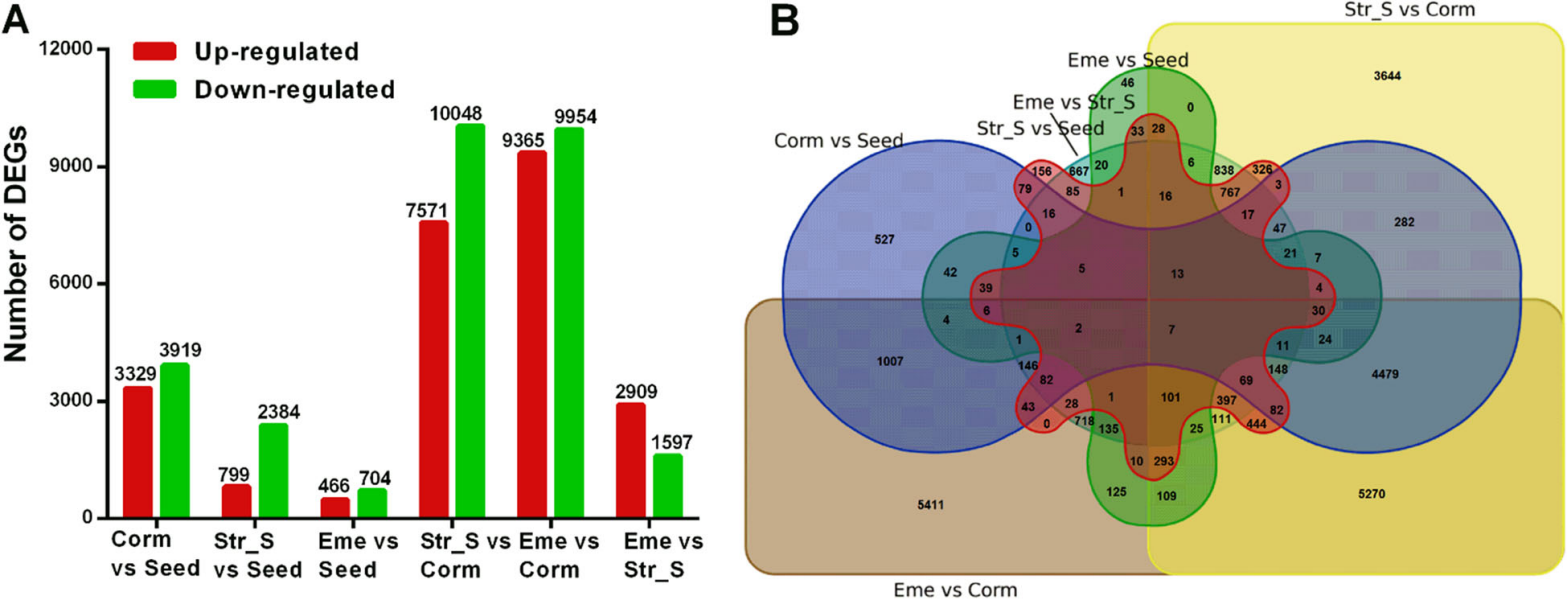
Pairwise comparisons revealed differentially expressed genes among four *P. sibiricum* seed stages*.* **A** Number of DEGs between two stages. Up-regulated and down-regulated refer to the expression level in the later stage of the comparison relative to the corresponding expression level in the earlier stage; **B** Venn diagram presenting the number of DEGs shared or unique among six comparisons

To investigate the gene expression dynamics and patterns in *P. sibiricum* seeds during warm and cold stratifications and seedling establishment, four stages (Seed, Corm, Str_S, and Eme; Fig. 1) were analyzed by RNA-seq using the Illumina PE150 platform, with three biological replicates for each stage. A total of 46–102 million clean reads were generated, of which 53.97–76.34% were mapped to SMRT reference transcripts using the RSEM software (Table S7). Gene transcription level was assessed using FPKM value (i.e., expected number of fragments per kilobase of transcript sequence per million base pairs sequenced), which adjusted the transcript length and sequencing depth. The data indicated that 30.84–40.01% of the genes in 12 samples had FPKM values < 0.1, whereas 32.71–39.86% of the genes had FPKM values > 1(Figure S4A). Unigenes with FPKM values > 0.3 were considered to be expressed. Accordingly, 62,984 genes were expressed in at least one of the 12 samples. The FPKM density distribution of the genes indicated that the overall gene expression of the Corm stage differed from that of the other three stages (Seed, Str_S, and Eme) (Figure S4B). The FPKM-based PCA confirmed that different *P. sibiricum* seed developmental stages were well separated, even though the three replicates for the Seed sample were more poorly clustered than the replicates for the other three stages (Figure S5A). A Pearson correlation analysis also revealed that the correlation between Seed2 and the Seed1 and Seed3 replicates (R^2^ = 0.68 and 0.66) (Figure S5B) was lower than the correlation among replicates for the other three stages. Therefore, only Seed1 and Seed3 were used for analyzing differential gene expression. Pairwise comparisons revealed that *P. sibiricum* seeds at the Corm stage had substantially more differentially expressed genes (DEGs), especially compared with the seeds at the Str_S and Eme stages (Fig. 3A). Using an adjusted *p* value < 0.05 and |log_2_ (fold-change)| ≥ 1 as the criteria, 7248, 17,619, and 19,319 DEGs were respectively detected in comparisons between the Corm stage and the Seed, Str_S, and Eme stages. Only 3183 and 1170 unigenes were differentially expressed between the Eme and Seed stages and between the Str_S and Seed stages, respectively. These results imply that imbibed *P. sibiricum* seeds with undeveloped embryos had physiological activities that were similar to those of *P. sibiricum* cold-stratified seeds and seedlings after the dormancy constraints in the Corm stage seeds were eliminated or alleviated by cold stratification. Of 27,059 DEGs, 46–5411 were differentially expressed only between two stages. Additionally, 8825 and 6428 DEGs were detected in two and three comparisons, respectively (Fig. 3B).

**Fig. 4:**
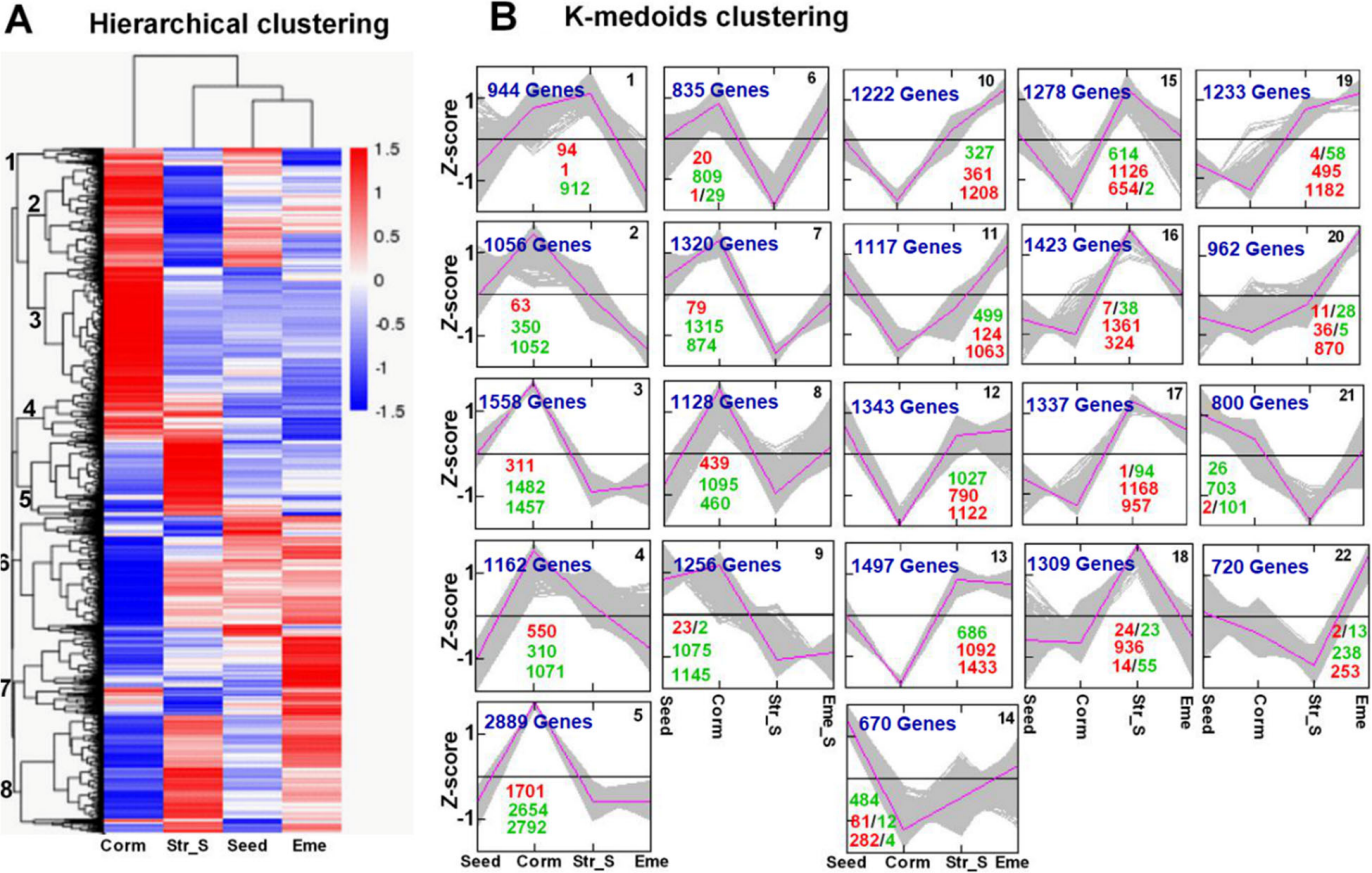
Expression patterns of *P. sibiricum* DEGs during seed dormancy release and seedling establishment. **A** Hierarchical clustering of all DEGs. Red and blue represent increased and decreased transcript abundances, respectively. The eight main subclusters are indicated at the branching points (left side). **B** Results of the K-medoids clustering. The *Z*-scores of log_2_ (FPKM+ 1) were used for the K-medoids clustering with the MEV software (v. 4.9.0) and Pearson correlations. The subcluster number is provided in the upper right corner of each subgraph. The numbers in dark blue indicate the number of DEGs in each subcluster. The numbers of DEGs in the Corm vs Seed, Str_S vs Corm, and Eme vs Corm comparisons are summarized (top to bottom) under the expression curves for each subcluster. Red and green numbers represent the number of transcripts with increased and decreased abundances, respectively

A hierarchical clustering analysis (Fig. 4A) classified 27,059 DEGs into eight main subclusters. Compared with the expression levels in the Seed, Str_S, and Eme stages, most genes in Subclusters 1–4 were more highly expressed at the Corm stage, whereas most genes in Subclusters 5–8 were expressed at lower levels. A subsequent K-medoids analysis (Fig. 4B) classified 27,059 DEGs into 22 subclusters. The expression of the DEGs in Subclusters 1–9 increased mainly at the Corm stage during a warm stratification and then decreased at the end of cold stratification or during seedling emergence, whereas the expression of the DEGs in Subclusters 10–22 generally had the opposite pattern. Specifically, Subclusters 1–8 included 3257 DEGs with up-regulated expression in the Corm vs Seed comparison, whereas Subclusters 10–15 comprised 3637 DEGs with down-regulated expression in the Corm vs Seed comparison. Moreover, 7571 and 10,048 DEGs with up- and down-regulated expression, respectively, were detected in the Str_S vs Corm comparison: 7543 of the 7571 DEGs were in Subclusters 10–19 (except Subcluster 14) and 9090 of the 10,048 DEGs were in Subclusters 2–8. Furthermore, 9763 DEGs with down-regulated expression in the Eme vs Corm comparison were in Subclusters 1–9 (except for Subcluster 6), whereas 9348 DEGs with up-regulated expression were in Subclusters 10–22 (except for Subclusters 18 and 21).

**Fig. 5:**
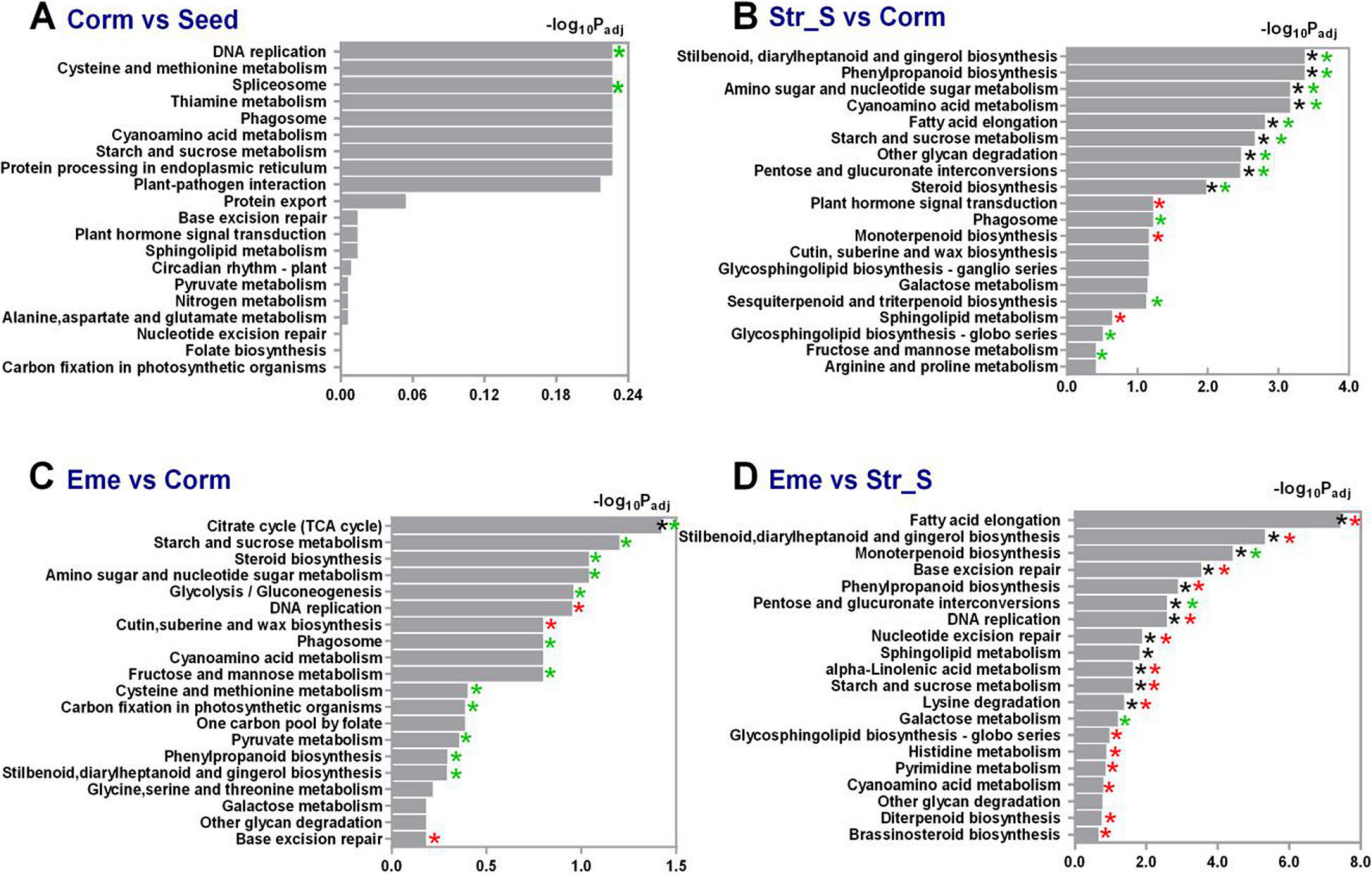
KEGG pathway enrichment analyses of *P. sibiricum* DEGs between Seed and Corm (**A**), Corm and Str_S (**B**), Corm and Eme (**C**), and Eme and Str_S **(D**) stages. A KEGG enrichment map was constructed based on the top 20 pathways and − log_10_ (adjusted *p* value) of the “all DEGs” sets in each comparison. *, *, and * (adjusted *p* value < 0.05) indicates the pathway was significantly enriched for the “all DEGs,” “up-regulated,” and “down-regulated” sets, respectively

The KEGG pathway enrichment analyses of the *P. sibiricum* DEGs in the Corm vs Seed, Str_S vs Corm, Eme vs Corm, and Eme vs Str_S comparisons involved the top 20 pathways and − log_10_ (adjusted *p* value) of “all DEGs” sets in each comparison (Fig. 5). The enriched pathways among the top 20 KEGG pathways differed greatly among the Str_S vs Corm, Eme vs Corm, and Eme vs Str_S comparisons. Only two KEGG pathways, “DNA replication” and “Spliceosome,” were enriched for the “all DEGs” set in the Corm vs Seed comparison (Fig. 5A). Many enriched KEGG pathways were identified for the “all DEGs” set and for the “down-regulated” gene set in the Str_S vs Corm comparison (Fig. 5B) or for the “up-regulated” gene set in the Eme vs Str_S comparison (Fig. 5D). Thirteen KEGG pathways, including “starch and sucrose metabolism,” were enriched for the “down-regulated” gene set, whereas three KEGG pathways, including “plant signal hormone transduction,” were enriched for the “up-regulated” gene set in the Str_S vs Corm comparison (Fig. 5B). Additionally, 15 KEGG pathways were significantly enriched for the “up-regulated” gene set, but only three enriched KEGG pathways were identified for the “down-regulated” gene set in the Eme vs Str_S comparison (Fig. 5D). The enriched KEGG pathways in the Eme vs Corm comparison, including “starch and sucrose metabolism” and “steroid biosynthesis,” were mainly associated with the “down-regulated” gene set (Fig. 5C).

### Expression of hormone metabolism and signaling genes during *P. sibiricum* seed dormancy and germination

**Fig. 6:**
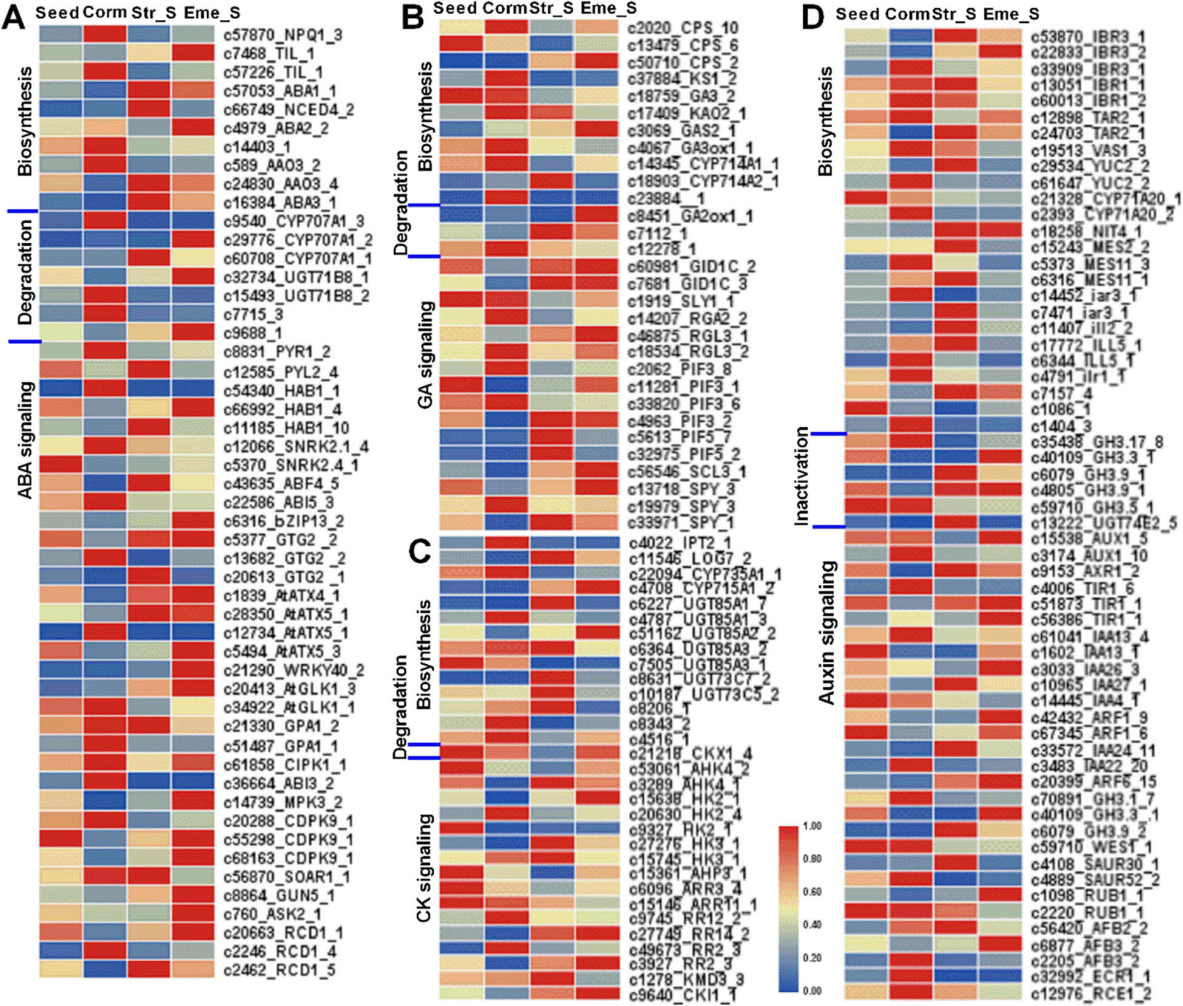
Representative expression patterns of *P. sibiricum* DEGs involved in ABA (**A**), GA (**B**), CK (**C**), and auxin (**D**) metabolism and signal transduction pathways. To calculate the relative expression level of a gene, its FPKM value was divided by the maximum FPKM value for that gene over four sampling stages. Heatmaps of selected DEGs were constructed using TBools without gene or group clustering. The genes are listed based on their positions in the hormone metabolism and signal transduction pathways. Partial IDs and abbreviated gene names as well as the number of DEGs in the same enzyme or functional family with similar expression profiles are presented on the right. Specific details are provided in Table S8

**Fig. 7:**
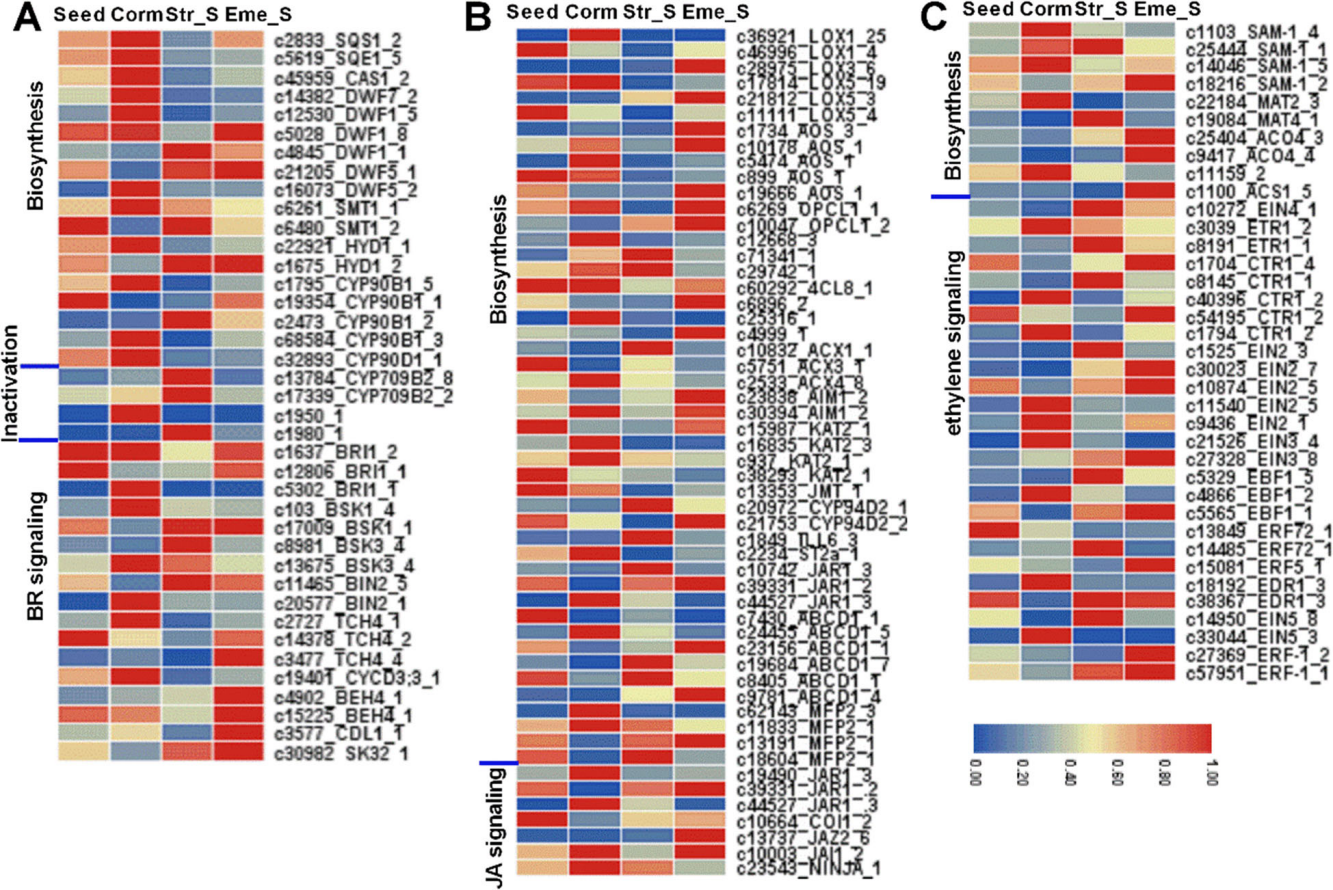
Representative expression patterns of *P. sibiricum* DEGs involved in BR (**A**), JA (**B**), and ethylene (**C**) metabolism and signal transduction pathways. To calculate the expression level of a gene, its FPKM value was divided by the maximum FPKM value for that gene over four sampling stages. Heatmaps of selected DEGs were constructed using TBools without gene or group clustering. The genes are listed based on their positions in the hormone metabolism and signal transduction pathways. Partial IDs and abbreviated gene names as well as the number of DEGs in the same enzyme or functional family with similar expression profiles are presented on the right. Specific details are provided in Table S8

A BLASTx search of the Arabidopsis protein database identified 1189 *P. sibiricum* unigenes possibly involved in the metabolism of ABA, GA, auxin, BR, and other hormones or the upstream pathways (Table S8). Additionally, 1285 putative hormone signaling genes were identified based on the KEGG annotations and published relevant information for Arabidopsis (Table S8). The expression levels of 475 hormone metabolism genes and 510 hormone signaling genes changed during *P. sibiricum* seed dormancy release and seedling emergence (Table S8, Figure S6). Figures 6 and 7 present the expression levels of the selected DEGs involved in the biosynthesis, degradation, and signaling of ABA (51), GA (30), CK (30), auxin (60), BR (39), JA (54), and ethylene (37) in four samples. The expression levels of many ABA signaling genes (13 of 34) were up-regulated in the Corm, Str_S, and Eme stages (Fig. 6A), reflecting the importance of ABA signaling for seed germination and dormancy release. The *CYP707A1* gene, which is involved in ABA degradation, was more highly expressed in the Corm (c9540), Str_S (c60708), and Eme (c29776) stages than in the Seed stage. The expression of a *GA3ox* gene (c4067) involved in GA biosynthesis was up-regulated in the Seed and Corm stages, especially compared with that in the Eme stage, whereas the expression of a *GA2ox* gene (c8451) involved in GA degradation was up-regulated in the Corm and Eme stages (Fig. 6B).

Fifteen ABA- and GA-related genes were selected for a quantitative real-time polymerase chain reaction (qRT-PCR) analysis to verify the accuracy of the RNA-seq-based expression levels during the *P. sibiricum* seed dormancy release process (Figures S7 and S8). We analyzed four seed dormancy release stages, three involving a stratification at 25 °C (Seed, Corm, and Eme) and one low-temperature stage (Str_S). The FPKM values indicated that two ABA biosynthesis-related genes, *ZEP* (zeaxanthin epoxidase) and *AAO3* (abscisic-aldehyde oxidase 3), and one ABA degradation-related gene (*CYP707A*) had consistent expression trends in the four samples. More specifically, *ZEP* and *CYP707A* were expressed at low levels in the Corm stage, in contrast to the relatively high expression level at the low-temperature stage (Str_S). These results indicate that an exposure to low temperatures is important for breaking the *P. sibiricum* epicotyl dormancy, which is required to complete the seed epitcotyl dormancy release process. The ABA signaling genes, including *ABI5* [basic leucine zipper (bZIP) transcription factor (TF)], *ABF* (ABA-responsive element-binding factor), *PYL4* (polyketide cyclase/dehydrase and lipid transport superfamily protein), *PYL8*, and *PP2C* (protein phosphatase 2C), were also analyzed by qRT-PCR. Their RT-qPCR results were basically consistent with the RNA-seq data. Two GA synthesis genes [*GA3ox* and *GAMT2* (gibberellic acid methyltransferase 2)], five GA signaling genes [*GASA3* (gibberellin-regulated protein 3), *GASA6*, *GASA14*, *GID1*, and *GID1-like* (gibberellin receptor)], and *CIGR2* (chitin-inducible gibberellin-responsive protein 2) also showed the consistent qRT-PCR and FPKM values (Figure S8). The *GA3ox* expression level was up-regulated at the Corm stage, whereas *GAMT2* expression was up-regulated at the Seed and Eme stages. The *GASA3*, *GASA6*, and *GASA14* genes were differentially expressed. Moreover, *GID1* and *CIGR2* were expressed at low levels during the Seed and Corm stages, but were highly expressed at the Str_S and Eme stages.

### Expression of transcription factors during *P. sibiricum* seed dormancy and germination

**Fig. 8:**
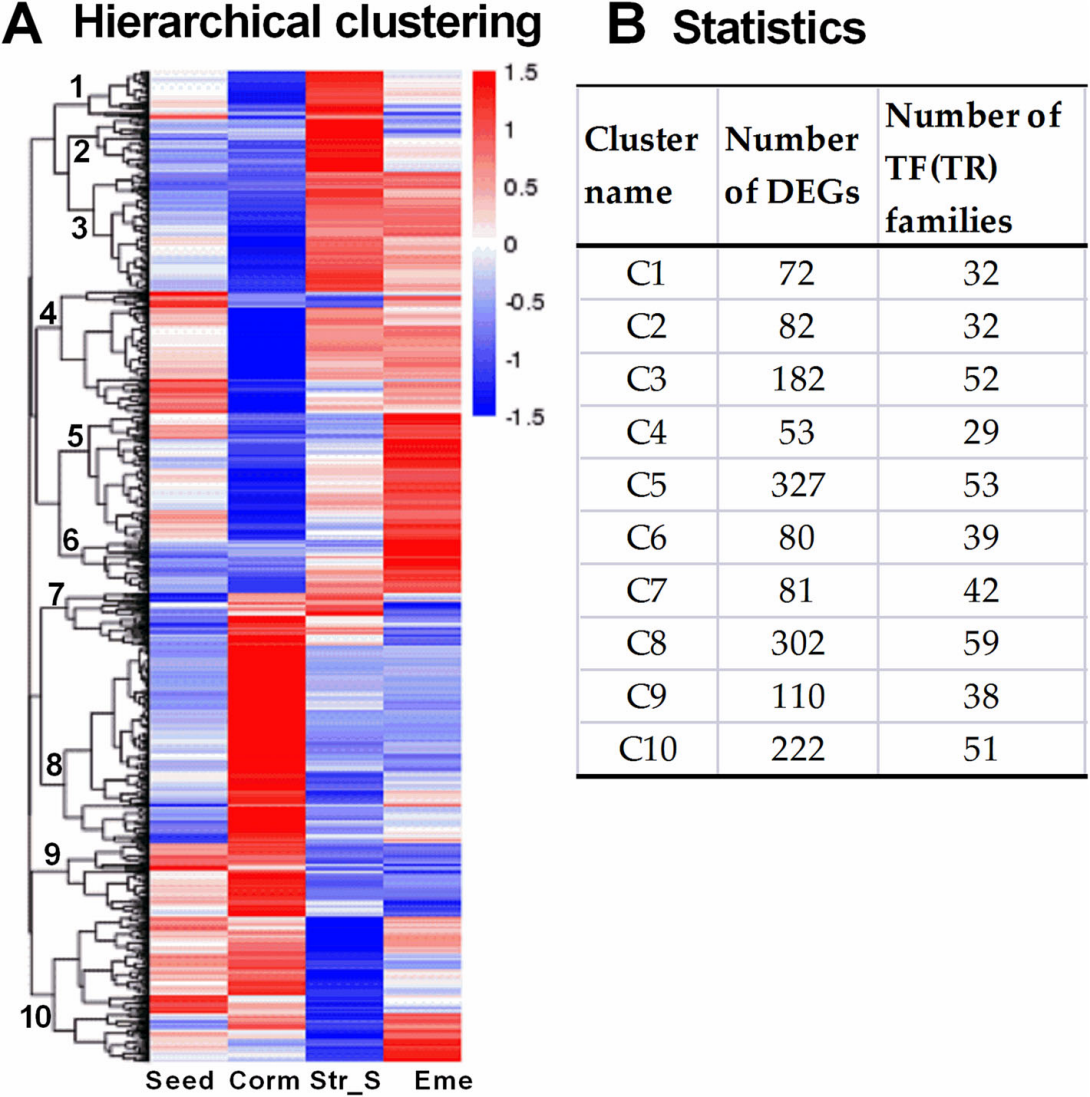
Expression patterns of differentially expressed TF and TR genes during seed dormancy release and seedling establishment. **A** Hierarchical clustering. Red and blue represent increased and decreased transcript abundances, respectively. The 10 main subclusters are indicated at the branching points (left side). **B** Statistics regarding the TF and TR DEGs in Clusters 1–10

Transcription factors are critical for seed development and germination [21–23]. In this study, we annotated our transcriptome using the iTAK software and the associated database, ultimately identifying 2605 TFs from 67 TF families as well as 1552 transcriptional regulators (TRs) from 25 TR families (Tables S9, S10). Among them, 1018 TFs (57 TF families) and 519 TRs (21 TR families) were differentially expressed during *P. sibiricum* seed stratification and germination. A hierarchical clustering analysis performed using the FPKM values of the TF and TR DEGs produced 10 subclusters (Fig. 8 and Table S9). These 10 subclusters were classified into two main groups based on the relative expression levels in the Corm samples: Subcluster 1–6 (542 TFs/ 273 TRs) in Corm with decreased expression while Subcluster 7–10 (476 TFs/246 TRs) having higher expression. -Some Arabidopsis TF/TR genes were functionally known to play roles in seed dormancy and germination (Table S11). The possible roles of the differentially expressed TFs and TRs are listed in Table S11 and are described in more detail below.

### Analysis of *P. sibiricum* DEGs related to seed dormancy release based on known Arabidopsis genes

**Fig. 9:**
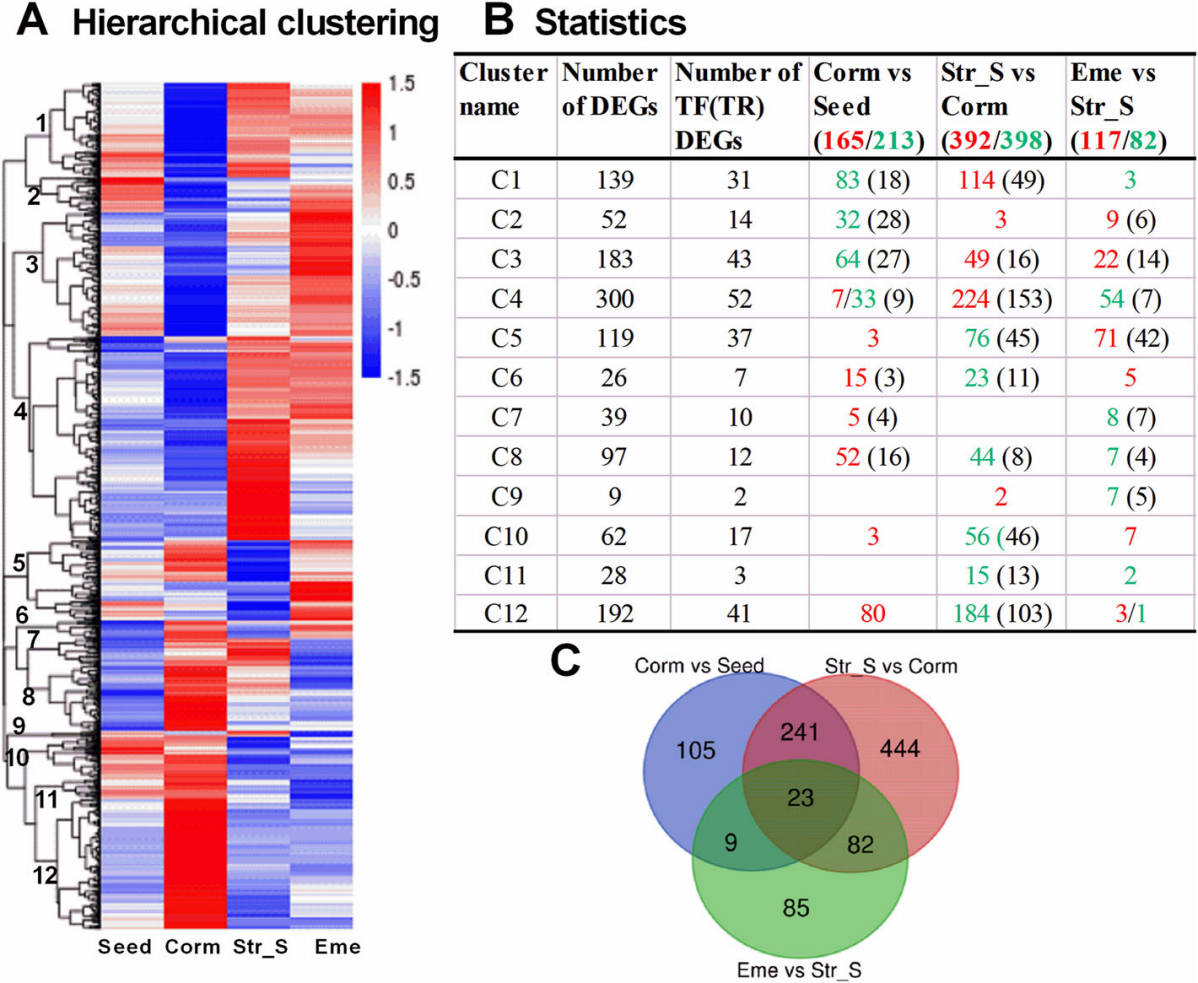
Expression patterns of differentially expressed seed dormancy-related genes during seed dormancy release and seedling establishment. **A** Hierarchical clustering. Red and blue represent increased and decreased transcript abundances, respectively. The 12 main subclusters are indicated at the branching points (left side). **B** Statistics of the DEGs in Clusters 1–12. The numbers of up-regulated DEGs (red) and down-regulated DEGs (green) in the Corm vs Seed, Str_S vs Corm, and Eme vs Str_S comparisons are listed in each subcluster. The numbers in parentheses refer to the number of DEGs exclusive to *P. sibiricum* MD release, PD release, and the seedling establishment after the MPD release. **C** Venn diagram presenting the number of shared or unique seed dormancy-related genes differentially expressed in the Corm vs Seed, Str_S vs Corm, and Eme vs Str_S comparisons

To date, a total of 630 Arabidopsis genes have been functionally verified to be involved in seed dormancy and germination (Table S11). A BLASTx search identified that 3407 *P. sibiricum* unigenes, including 688 encoding TFs or TRs are homologs of 413 of these known Arabidopsis genes. An analysis of differential gene expression based on the FPKM values of four samples identified 1246 DEGs, including 269 TF/TR genes. Among them, 378, 790, and 199 DEGs were associated with *P. sibiricum* MD release (Corm vs Seed), PD release (Str_S vs Corm), and the seedling establishment after the MPD release (Eme vs Str_S) (Table S11, Fig. 9). A hierarchical clustering analysis using the FPKM values of these 1246 DEGs resulted in 12 subclusters (Fig. 9A). The expression of 674 DEGs in Subclusters 1–4 (140 TFs/TRs) was down-regulated mainly in the Corm samples, but was up-regulated in the Str_S samples. The expression levels varied in the Seed and Eme samples. Additionally, we detected 212 down-regulated DEGs in the Corm vs Seed comparison, 390 up-regulated DEGs in the Str_S vs Corm comparison, and 31/57 up/down-regulated DEGs in the Eme vs Str_S comparison (Fig. 9B). Subclusters 5–12 (Table S11) contained 572 DEGs (129 TFs/TRs), with up-regulated expression mainly in the Corm samples, but down-regulated expression in the other three samples. Furthermore, 158 up-regulated DEGs in the Corm vs Seed comparison, 398 down-regulated DEGs in the Str_S vs Corm comparison, and 108/25 up/down-regulated DEGs in the Eme vs Str_S comparison were distributed in these eight subclusters (Fig. 9B). Of the DEGs identified by the Corm vs Seed, Str_S vs Corm, and Eme vs Str_S comparisons, only 23 were identified in all three comparisons. We detected 105, 444, and 85 DEGs that were specifically expressed during *P. sibiricum* MD release, PD release, and the seedling establishment after the MPD release, respectively. A Venn diagram analysis revealed 241, 82 and 9 DEGs common to the Corm vs Seed and Str_S vs Corm comparisons, the Str_S vs Corm and Eme vs Str_S comparisons, and the Corm vs Seed and Eme vs Str_S comparisons, respectively (Fig. 9C). The DEGs unique to *P. sibiricum* MD release were distributed in Subclusters 1–4, and 6–8. In contrast, the DEGs unique to *P. sibiricum* PD release were distributed in nine subclusters except Subcluster 2, 7, 9. The DEGs unique to the seedling establishment after the MPD release were distributed in Subclusters 2–5, and 7–9 (Fig. 9B).

The known functions of Arabidopsis genes involved in seed dormancy and germination can be classified into several categories, including hormone/light signal transduction, calcium-mediated signaling, chromatin remodeling, mRNA degradation, stress response (*AtCYS5*), organ development (*AtHYL1*/*AtSE*/*AtDCL1*/*ATGSO*), and storage matter metabolism (*AtATE1*/*AtATE2*/*AtPRT6*) (Table 2; Table S11). Subclusters 5–12 included ABA signaling and regulatory genes (e.g., *ABF1*, *ABI3*, *ABI5*, *SIZ1*, and *SUA*), GA signaling genes [e.g., *LEUNIG_HOMOLOG* (*LUH*), *RGI3*, *SYL1*, and *SPY*], CK signaling genes (e.g., cytokinin receptor genes *AHK2* and *AHK4*), auxin signaling and responsive genes [*AUXIN RESPONSE FACTOR* (*ARF2*), *AUXIN RESISTANT1* (*AUX1*), *IAA8*, *LAX*, and *PIN*], BR signaling genes (*BIN2* and *BRI1*), calcium-mediated signaling genes (e.g., *CAM7*, *CAMAT6*, *CIPK3*, *CPK6*, and *CML39*), genes integrating multiple signals [*FAR-RED IMPAIRED RESPONSE1* (FAR1), *GIGANTEA* (*GI*), *MYC2*, and *TZF1* (tandem zinc finger)], chromatin remodeling genes [e.g., *CHR12* (chromatin remodeling ATPase), *HAC1* (histone acyltransferase), *HDA19* (histone deacetylase), *PICKLE* (chromatin-remodeling factor), *SUVH5* (histone methyltransferase), *ROS1* (DNA demethylase), and *POWERDRESS* (epigenetic factor)], cell wall loosening expansin genes (*EXPA4* and *EXPA8*), and seed storage matter metabolism-related genes [*CathB3* (cathepsin B-like cysteine protease), *MAN2* (endo-β-mannanase), *MAN7*, *PROTEOLYSIS6*, and *ANNAT2* (annexin A2)]. Subclusters 1–4 included genes with the same functions as some of the genes in Subclusters 5–12 (e.g., *ARF2* and *AHK4*) as well as genes that were exclusive to these four subclusters [e.g., *AREB3*, *CORONATINE INSENSITIVE 1*, *GID1* (GA receptor), and *GOLS1* (galactinol synthase)]. The expression of some DEGs that were annotated as the same Arabidopsis homolog or gene family exhibited diverse expression patterns depending on the stage, suggesting they have different roles during *P. sibiricum* MD release, PD release, and the seedling establishment after the MPD release, and are therefore regulated differently. For example, 26 DEGs were annotated as *AtWRKY2*, *6*, *40*, and *41*. The expression levels of 11 WRKY TF genes in Subclusters 3 and 5 were all slightly down-regulated during MD release and fluctuated during the cold stratification; however, the expression levels of all of these genes were significantly up-regulated at the seedling establishment stage (relative to the expression levels in the MPD seeds). The expression of 15 DEGs annotated as *AtWRKY2* in Subclusters 4 (1), 8 (1), 10 (4), 11 (1), and 12 (5) exhibited distinct changing trends during MPD release and seedling establishment. The expression of *AtWRKY6*, which encodes a positive regulator of ABA signaling during seed germination and early seedling development, is reportedly repressed during seed germination [24], but it is substantially up-regulated in Arabidopsis germinated seeds under light [25] (http://bar.utoronto.ca/efp/cgi-bin/efpWeb.cgi). However, AtWRKY2 mediates ABA-dependent seed germination and early postgermination growth [26], and *AtWRKY2* expression is up-regulated considerably by exogenous ABA [26] and in germinated seeds under light [25] (http://bar.utoronto.ca/efp/cgi-bin/efpWeb.cgi). In our study, most *P. sibiricum* homologs of *AtWRKY2* were slightly or significantly more highly expressed in the corm than in the seed, but the expression levels decreased during PD release and seedling emergence, which may be closely related to the simultaneous decrease in the expression of its regulators ABI5 and ABI3 [26].

**Table 2:** Functions and fold changes of selected seed dormany/germination-related DEGs during MD and PD release

Unigene ID	Gene name	Function	Corm vs Seed	Str_S vs Corm	Eme vs Str_S
i0_LQ_SeedM_c3069/f1p14/781	AtGIM2	GA biosynthesis	1.85		
i1_HQ_SeedM_c1802/f5p2/1957	AtNPF3.1	GA transport	−1.76	2.63	−3.78
i0_LQ_SeedM_c1919/f2p0/830	AtSLY1	GA signaling		−2.23	
i3_LQ_SeedM_c33971/f1p0/3810	AtSPY	GA signaling	−3.75	3.98	
i4_LQ_SeedM_c21157/f1p3/4409	AtSPY	GA signaling	2.66	−1.96	
i1_HQ_SeedM_c1756/f2p5/1840	AtGID1c	GA signaling	−1.53	2.46	
i2_LQ_SeedM_c18534/f1p7/2257	AtRGI3	GA signaling		−1.47	
i2_HQ_SeedM_c14207/f11p9/2283	AtGAI	GA signaling		−2.8	
i0_LQ_SeedM_c8549/f1p3/650	AtGASA6	GA signaling		−7.4	4.14
i1_LQ_SeedM_c9540/f1p1/1818	AtCYP707A1	ABA catabolism	3.28	−5.32	
i1_LQ_SeedM_c24968/f1p3/1093	AtPYL8	ABA signaling		1.46	
i0_LQ_SeedM_c8831/f1p3/976	AtPYR1	ABA signaling		−2.4	
i3_LQ_SeedM_c18408/f1p3/3496	AtS2P	ABA signaling	3.3	−2.78	
i2_LQ_SeedM_c46616/f3p4/2678	AtHAB1	ABA signaling	−1.67	1.43	
i1_LQ_SeedM_c3858/f2p106/1553	AtHON	ABA signaling	−2.52	3.13	
i2_LQ_SeedM_c25671/f1p3/2973	AtAFP3	ABA signaling	−2.4	1.94	
i1_LQ_SeedM_c5687/f1p17/2029	AtPP2CA	ABA signaling		2.62	
i1_LQ_SeedM_c9533/f1p0/1988	AtPP2C5	ABA signaling	−1.81		2.65
i3_LQ_SeedM_c20125/f1p4/3251	AtSnRK2.6	ABA signaling		−2.63	
i0_LQ_SeedM_c10659/f1p0/461	AtSnRK3.6	ABA signaling		−2.7	
i5_LQ_SeedM_c2274/f1p2/5125	AtPP2CE	EGR2 phosphatase	−3.34	2.56	
i1_LQ_SeedM_c6124/f1p7/1511	AtHAI3	ABA signaling	−1.75	2.01	
i4_LQ_SeedM_c21538/f1p0/4527	AtABF4	ABA signaling		2.9	
i1_LQ_SeedM_c8820/f1p3/1489	AtWRKY41	regulator of ABI3 expression		−2.5	4.43
i2_LQ_SeedM_c36664/f1p0/2863	AtABI3	ABA signaling		−6.26	
i1_LQ_SeedM_c22586/f1p9/1509	AtABI5	ABA signaling		−1.89	
i3_HQ_SeedM_c17466/f2p2/3841	AtARF2	Auxin signaling	−5.55	7.14	−1.87
i3_LQ_SeedM_c7465/f1p21/3129	AtARF2	Auxin signaling	2.21	−4.07	
i0_HQ_SeedM_c12179/f2p3/788	AtIAA8	Auxin signaling		−1.83	
i2_LQ_SeedM_c3798/f1p9/2925	AtAUX1	auxin transport	4.36	−3.19	
i3_LQ_SeedM_c14993/f1p0/3065	AtAUX1	auxin transport	−3.58	3.13	
i2_LQ_SeedM_c41269/f1p5/2679	AtLAX	auxin transport	2.13	−3.86	
i4_LQ_SeedM_c20675/f1p4/4170	AtABCB1/PGP1	auxin transport	2.49	−5.04	
i4_LQ_SeedM_c4985/f1p0/4218	AtABCB1/PGP1	auxin transport	−4.58	3	
i4_LQ_SeedM_c3741/f1p0/4833	AtABCB19/PGP19	auxin transport	2.77	−2.41	−1.92
i4_LQ_SeedM_c18157/f1p5/4494	AtABCG36	auxin transport	−3.28	6.15	−3.33
i2_HQ_SeedM_c17187/f5p6/2691	AtPIN1	auxin transport	−1.61	2.1	
i2_LQ_SeedM_c11796/f1p4/2422	AtPIN1	auxin transport	4.76	−6.81	
i2_LQ_SeedM_c37994/f1p3/2426	AtPIN1	auxin transport		−2.99	2.73
i2_LQ_SeedM_c41403/f1p2/2390	AtPIN2	auxin transport		−2.9	1.88
i2_LQ_SeedM_c56557/f1p2/2726	AtPIN3	auxin transport		1.85	
i2_LQ_SeedM_c60075/f1p3/2081	AtPIN4	auxin transport		−5.78	
i1_HQ_SeedM_c2547/f4p0/2003	AtNRT1.1	auxin transport	5.38	− 4.32	
i2_LQ_SeedM_c37693/f1p22/2372	AtBIN2	BR signaling	−2.05	2.97	
i1_LQ_SeedM_c20577/f1p2/1956	AtBIN2	BR signaling	5.57		
i3_HQ_SeedM_c11682/f2p0/3684	AtBRI1	BR signaling	−6.27	5.35	
i3_LQ_SeedM_c5302/f1p0/3667	AtBRI1	BR signaling	5.44	−6.97	
i1_LQ_SeedM_c10003/f1p34/1668	AtMYC2	JA signaling		−1.92	
i2_LQ_SeedM_c56779/f1p3/2496	AtAKIN10	Energy sensor	−2.81	2.76	
i2_HQ_SeedM_c26414/f2p8/2462	AtCOI1	ubiquitin-dependent protein degradation		1.22	
i0_LQ_SeedM_c9980/f1p0/847	AtASK2	ubiquitin-dependent protein degradation	−2.8	2.15	
i0_LQ_SeedM_c3002/f1p6/571	AtRHA2b	ubiquitin-dependent protein degradation		−2.56	
i3_HQ_SeedM_c7511/f2p11/3746	AtUBP26	ubiquitin-dependent protein degradation	2.92	−2.44	
i2_LQ_SeedM_c48471/f2p3/2487	AtATE2	protein degradation		−1.91	
i4_HQ_SeedM_c15243/f2p0/4126	AtCOP10	ubiquitin-conjugating enzyme E2		−1.58	
i3_LQ_SeedM_c15448/f1p3/3019	AtDWA	substrate receptor for cullin-RING ubiquitin ligase 4 complexes for protein degradation	−2.11	1.95	
i0_LQ_SeedM_c5286/f1p0/992	AtCathB3	Cysteine proteinase	4	−4.3	
i3_LQ_SeedM_c32927/f1p20/3736	AtSIZ1	protein sumoylation	3.27	−4.75	
i3_LQ_SeedM_c22466/f1p6/3768	AtSIZ1	protein sumoylation	−1.89	2.66	
i2_LQ_SeedM_c11836/f4p3/2198	AtKAPP	kinase-associated protein phosphatase	3.34	−4.77	
i2_LQ_SeedM_c56819/f1p3/2379	AtKAPP	kinase-associated protein phosphatase	−4.31	3.14	
i1_LQ_SeedM_c7267/f1p11/1962	AtDjA3	Chaperone protein	−3.28	2.05	
i2_LQ_SeedM_c66006/f1p10/2500	AtDjA3	Chaperone protein	2.34	−2.54	
i0_LQ_SeedM_c17946/f1p1/823	AtCAM7	calcium signaling	1.76	−3.25	
i4_LQ_SeedM_c16211/f1p0/4095	AtCAM7	calcium signaling	−4.25	3.63	
i1_LQ_SeedM_c15573/f1p3/1075	AtCML39	calcium signaling		−4.27	6.47
i4_LQ_SeedM_c16133/f1p0/4048	AtCAMTA6	calmodulin-binding TF		3.78	
i3_LQ_SeedM_c4760/f1p1/3504	AtCAMTA6	calmodulin-binding TF		−2.51	
i2_LQ_SeedM_c52294/f1p1/2157	AtIQM4	Ca2 + −independent CaM-binding protein		1.69	
i2_LQ_SeedM_c59734/f2p3/2262	AtCPK6	Calcium-dependent protein kinase	4.13	−3.16	
i3_LQ_SeedM_c18100/f1p6/3246	AtGLR3.5	ionotropic glutamate receptor	−1.9		
i1_LQ_SeedM_c21031/f1p0/1778	AtCAT1	catalase	4.88	−7.79	4.03
i1_LQ_SeedM_c5525/f1p5/1792	AtTFIIS	transcript elongation factor		−1.59	1.53
i2_HQ_SeedM_c3213/f3p5/2849	AtCBC80	RNA metabolism	−3.02		
i2_LQ_SeedM_c13505/f1p1/2439	AtCFM9	RNA metabolism		−1.82	
i6_LQ_SeedM_c1081/f1p4/6064	AtDCL1	RNA metabolism	2.82	−2.07	−2.58
i2_LQ_SeedM_c23333/f1p14/2786	AtSE	RNA metabolism	−2.41	2.59	
i2_LQ_SeedM_c27773/f1p0/2851	AtNG1	RNA metabolism		2.71	
i0_LQ_SeedM_c2368/f1p9/737	AtGRP7	RNA metabolism	−4	3.68	
i4_HQ_SeedM_c3551/f2p0/4808	AtSUA	RNA metabolism	−6.6	5.58	
i3_LQ_SeedM_c23460/f1p11/3454	AtSUA	RNA metabolism	6.77	−6.94	
i2_LQ_SeedM_c23171/f1p11/2948	AtFY	mRNA processing	2.17	−2.07	
i3_LQ_SeedM_c20086/f1p0/3799	AtFY	mRNA processing	−4.59	4.24	
i3_LQ_SeedM_c30639/f1p0/3050	AtSF1	mRNA processing	6.59	−7.57	
i3_LQ_SeedM_c9329/f1p0/3408	AtSF1	mRNA processing		6.55	
i1_LQ_SeedM_c4043/f1p9/1960	AtRRP41L	mRNA degradation		1.74	
i3_LQ_SeedM_c9561/f1p61/3436	AtXRN4	mRNA degradation	4.36	−6.43	
i5_LQ_SeedM_c4155/f1p1/5195	AtXRN4	mRNA degradation		5.44	−5.8
i4_LQ_SeedM_c2767/f1p0/4467	AtVCS	mRNA degradation	−3.54	2.82	
i3_LQ_SeedM_c9026/f1p0/3591	AtVCS	mRNA degradation	1.95	−2.55	
i2_LQ_SeedM_c10778/f1p19/2311	AtTudor2	mRNA degradation	−1.55	1.58	
i1_LQ_SeedM_c2372/f1p11/1714	AtCHO1	AP2/ERF-AP2 TF		−2.13	
i2_LQ_SeedM_c29982/f1p4/2011	AtASG1	DNA helicase		−1.93	
i2_LQ_SeedM_c4618/f1p3/2576	AtASG1	DNA helicase		2.39	
i4_LQ_SeedM_c25313/f1p14/4107	AtEMF1	histone methylation	−2.76	2.38	
i5_LQ_SeedM_c10657/f1p1/5263	AtCHR12	chromatin modification	3.6	−3.5	
i3_LQ_SeedM_c6562/f1p0/3365	AtCLF	chromatin modification	−1.98	2.68	
i6_LQ_SeedM_c990/f1p0/6104	AtEFS	chromatin modification		−3.15	
i5_HQ_SeedM_c466/f6p0/6094	AtHAC1	chromatin modification	−2.19	1.83	
i4_LQ_SeedM_c1879/f1p8/5031	AtHAC1	chromatin modification	2.98	−2.54	
i5_LQ_SeedM_c8676/f1p0/5895	AtPKL	chromatin modification	−8.64	7.42	2.2
i4_LQ_SeedM_c13881/f1p0/4335	AtPKL	chromatin modification	1.98	−2.06	2.01
i3_LQ_SeedM_c14311/f1p0/3164	AtSUVH5	chromatin modification		−4.1	2.64
i3_LQ_SeedM_c6655/f1p1/3369	AtHDA9	chromatin modification		4.93	2.32
i2_LQ_SeedM_c6979/f1p2/2448	AtHDA15	chromatin modification		−1.56	
i4_LQ_SeedM_c18037/f1p21/4801	AtPWR	chromatin modification	1.93	−2.1	1.61
i2_LQ_SeedM_c27191/f1p4/2756	AtCRY1	light signaling	3.66	−3.76	
i2_HQ_SeedM_c35624/f2p2/2375	AtCRY2	light signaling	−2.16	3.56	
i3_LQ_SeedM_c33308/f1p0/3596	AtPHYA	light signaling	2.9	−4.08	
i2_LQ_SeedM_c36242/f1p4/2456	AtPIF3	light and GA signaling	−5.16	4.2	
i2_LQ_SeedM_c21496/f1p3/2439	AtPIF3	light and GA signaling		−2.43	
i1_LQ_SeedM_c17359/f1p1/1778	AtCIPK3	CBL-interacting protein kinase	−2.74	2.91	−2.3
i3_LQ_SeedM_c21126/f1p9/3152	AtPRR9	circadian rhythm	−7.9	6.9	
i2_LQ_SeedM_c52693/f1p0/2189	AtTOC1	circadian rhythm		1.7	
i2_LQ_SeedM_c48681/f1p5/2590	AtMED25	light and JA signaling	1.98	−1.94	
i1_LQ_SeedM_c10304/f1p0/1883	AtCYCD1;1	cell cycle protein		−2.59	1.73
i2_LQ_SeedM_c66356/f1p3/2916	AtSAUR62	Cell-elongated related genes		2.01	
i1_HQ_SeedM_c2317/f3p4/1843	AtWRKY2	WRKY TF	3.07	−10.19	
i2_LQ_SeedM_c69494/f1p7/2362	AtWRKY2	WRKY TF	−2.1	3.34	
i2_LQ_SeedM_c59966/f1p0/2048	AtWRKY6	WRKY TF		4.72	
i2_LQ_SeedM_c50026/f1p2/2377	AtWRKY40	WRKY TF		−2.47	4.13
i3_LQ_SeedM_c23462/f1p0/3258	AtGBF1	bZIP TF		2.1	
i2_LQ_SeedM_c36125/f1p5/3079	AtLUG	transcriptional corepressor	2.2	−5.54	
i3_LQ_SeedM_c29344/f1p0/3137	AtLUG	transcriptional corepressor	−3.02	3.29	
i2_LQ_SeedM_c21274/f1p0/2367	AtNIA1	nitrate assimilation	6.39	−7.04	
i3_LQ_SeedM_c30611/f1p0/3119	AtGCN2	amino acid metabolism	−7.11	6.39	
i1_LQ_SeedM_c17195/f2p0/1544	AtGln1;1	amino acid metabolism	1.47	−1.53	
i3_LQ_SeedM_c12339/f1p3/3098	AtTPS5	trehalose biosynthesis	−1.89		
i1_LQ_SeedM_c20939/f1p0/1223	AtGOLS1	galactinol synthase		4.59	−1.69
i2_HQ_SeedM_c62143/f13p14/2507	AtMFP2	Fatty acid degradation	3.92	−4.32	
i2_LQ_SeedM_c18604/f1p14/2485	AtMFP2	Fatty acid degradation		3.99	
i1_LQ_SeedM_c11103/f1p4/1439	AtLIP1	lipid metabolism	2.62	−1.9	1.69
i3_LQ_SeedM_c26493/f1p7/3651	AtKPNB1	protein transport		−1.58	
i3_LQ_SeedM_c30926/f1p0/3119	AtPHO1	phosphate ion transport		−4.63	
i2_LQ_SeedM_c20018/f1p30/2524	AtCER9	cuticular wax biosynthesis	−3.68		2.14
i1_LQ_SeedM_c5889/f1p2/1648	AtPGIP2	cell wall metabolism		−1.6	
i1_LQ_SeedM_c8885/f1p0/1852	AtPLY	cell wall metabolism	2.57	−5.77	
i1_LQ_SeedM_c22277/f1p18/1916	AtPME58	cell wall metabolism		5.59	−4.03
i1_LQ_SeedM_c20327/f1p9/1915	AtMAN2	cell wall metabolism		−8.51	5.39
i2_LQ_SeedM_c17702/f1p2/2902	AtMAN7	cell wall metabolism	3.28	−4.5	
i1_LQ_SeedM_c15435/f1p3/1132	AtEXPA4	cell wall metabolism	1.58	−3.82	1.66
i1_LQ_SeedM_c2665/f1p0/2002	AtNEK6	cortical microtubule organization		−1.92	
i2_LQ_SeedM_c6535/f2p1/2253	AtDREB2C	DREB TF		1.73	
i2_LQ_SeedM_c3709/f1p3/2689	AtERD15	dehydration-induced protein	−3.37	1.92	
i1_LQ_SeedM_c22084/f1p0/1316	AtLEA31	Seed maturation protein		4.12	−1.77
i4_LQ_SeedM_c19775/f1p33/4207	AtGSO1	peptide hormone signaling		−2.57	

Note: Gene was named based on annotated Arabidopsis homologs. The full information of gene regulating Arabidopsis seed dormancy and germination was given in Table S11

continues to grow and differentiate, which is accompanied by the emergence of a plumule

## Discussion

*Polygonatum sibiricum* is a well-known traditional Chinese medicinal plant throughout east Asian countries. Studies regarding the cultivation and seed biology of this plant species have been conducted, but its seed germination characteristics remain unclear [17, 27–30]. Cheng et al. and Zhu et al. reported that GA_3_ and a low-temperature treatment (0 °C for 120 days) may enhance *P. sibiricum* seed germination [28, 30]. However, our research [12] suggested that warm stratification followed by a low-temperature stratification is a more appropriate strategy for inducing *P. sibiricum* seed germination, which accords with the characteristics of the seeds with deep simple epicotyl MPD [31]. Under natural conditions, *P. sibiricum* mature seeds are dispersed in mid-fall and germinate under suitable warm conditions during the following spring and summer, with only the radicle and corm emerging. The shoot emerges in the spring after the second cold winter, indicating that *P. sibiricum* seeds exhibit the deep morphophysiological epicotyl dormancy. However*, P. sibiricum* seeds can develop into seedlings within 6 months following the successive exposures to warm/cold conditions [12]. *Polygonatum kingianum*, another important source of polygonati rhizoma, is mainly grown in Yunnan province, China. Its mature seed has an underdeveloped embryo and possesses the similar seed germination characteristics to *P. sibiricum* [32]*.* However, they had the distinct seedling establishment processes after corm formation. No *P. sibiricum* seedling emerged under prolonged warm conditions after corm formation, indicating that a cold treatment is essential for the epicotyl dormancy release of *P. sibiricum* corm and its leaf emergence. However, *P. kingianum* germinated seeds had about 50% seedling emergence when inculated at 25 °C [33]. In view of this, an additional low-temperature treatment seems unnecessary for *P. kingianum* seedling emergence after corm formation, although an appropriate chilling period can enhance the emergence rate of *P. kingianum* **s**eedling and accelerate its emergence [32, 33]. These results indicated that *Polygonatum* species growing in different climatic regions have distinct seed germination properties and regulatory mechanisms.

To understand the molecular mechanisms of corm formation under warm temperature, cold stratification to break the epicotyl dormancy, and seedling establishment during *P. sibiricum* seed germination, we performed gene expression analyses of four stages (Seed, Corm, Str_S, and Eme) using transcriptome sequencing techniques. During the MD release, *P. sibiricum* seeds stratified at warm temperature undergo several important morphological changes including embryo growth, radicle emergence and elongation, and formation of a plumule-containing cormlet. Our DEG analyses revealed that there were more DEGs in the Corm vs Str_S and Corm vs Eme comparisons than in the other comparisons, implying that corm development is a key stage for the transcriptional regulation of *P. sibiricum* seed germination and seedling emergence. It also indicated that a specific cold stratification treatment period breaks the epicotyl dormancy via the expression-level changes of many genes in the developed corm. Fewer DEGs between the mature seed and the corm were identified during MD release. This may have been because our sampling time-points with the bulk seed did not cover all the important developmental events occurring in embryo differentiation and endosperm weakening during MD release, given that the genes determining plant development and growth are usually spatially and temporally expressed [34, 35]. Wang et al. [32] found that 7 TFs including *DAG2*, *Dof5.7*, *bZIP60*, *MYB111*, *MYB55*, *MYB46*, and *REM1* possibly regulated the expression of 17 hub genes that were altered among three different dormant statuses of *P. kingianum* corm. However, we only detected three homologs of *AtbZIP60* in our experimental condition and found that they (c18106, c17463 and c8065) were all decreased during cold stratification and elevated in Corm and Eme under warm temperature (Table S9). *bZIP60* TF has been found to modulate the unfolded protein response (UPR) in plants and could enhance heat stress tolerance [36]. The upregulation of bZIP60 transcripts in *P. sibiricum* corm and seedling under warm temperature may suggest its role of warm temperature tolerance.

Studies on plant species with MPD seeds [37–40] revealed that their seed dormancy and germination is controlled jointly by endogenous hormones and environmental conditions including temperature, soil or seed moisture, light, smoke, and nutrient availability, which is similar to Arabidopsis and cereal crops with physiological dormant seeds [41–45]. In addition to ABA and GA, which are two major hormones that respectively induce and break seed dormancy in most plants, other phytohormones, such as cytokinins, jasmonic acid, strigolactones, brassinosteroids, ethylene, salicylic acid, and auxin, may also regulate seed dormancy and germination in a plant species-dependent manner. Their contents in dormant and germinating seeds are partly regulated by the expression of genes related to hormone metabolism and signaling [40, 46]. In this study, we identified 475 putative hormone metabolism-related unigenes and 510 putative hormone signaling genes that were differentially expressed during *P. sibiricum* seed dormancy and seedling and shoot emergence (Figs. 6 and 7, Figure S6). Although ABA is considered to be crucial for inducing and maintaining seed dormancy, most *P. sibiricum* DEGs regulating ABA biosynthesis and degradation such as NPQ1, ABA2, CYP707A1 were more highly expressed in the Corm, Str_S, and Eme stages than in the Seed stage. *ABI3* and *ABI5* are two ABA signaling-related genes that positively control seed dormancy [43]. ABI5 is found highly expressed in dormant seeds of several plant species [19, 46]. Their homologous genes in *P. sibiricum* were expressed at higher levels in the Seed and Corm stages than in the cold-stratified germinated seeds and seedlings, implying ABA may have accumulated during the Seed and Corm stages under warm conditions, leading to the dormancy of the Seed and Corm stages. In *P. cyrtonema*, which has similar seed dormancy/germination characteristics to *P. sibiricum*, the ABA level in seeds stored for a long period in wet sand is reportedly higher under warm conditions than under cold conditions [40]. It was also found that *P. kingianum* germinated seeds at corm stage had the higher ABA content than cold-stratified and non-dormant germinated seeds, which was consistent with the higher expression of ABA synthesis-related transcripts [33]. Fluridone is an inhibitor of ABA biosynthesis and promoted seed dormancy release like cold stratification [47, 48]. In our recent pre-experiments, we observed that fluridone-treated *P. sibiricum* seeds germinate at a higher rate than untreated seeds (data not shown). Hence, a fluridone treatment of *P. sibiricum* MD seeds and the corm after MD release may be useful for elucidating the effects of ABA on *P. sibiricum* seed MD release and the induction of epicotyl physiological dormancy under warm conditions as well as epicotyl dormancy under cold conditions.

Consistent with molecular regulation of PD seed germination of Arabidopsis [43], many genes related to chromatin modifiers and remodelers, DNA methylation, mRNA degradation, and cell wall structures were also differentially regulated during *P. sibiricum* seed germination, epicotyl dormancy release, and seedling establishment (Table 2, Fig. 9, Table S11). During the germination of *P. polyphylla*, *P. quinquefolius*, and *P. suffruticosa* seeds, the cotyledons remain inside the seed coat/endosperm after MD release and have to be pulled outside until the endosperm is sufficiently weakened to eliminate the mechanical resistance from the surrounding tissues (testa and endosperm). In contrast, the plumule of *P. sibiricum* develops outside of the hard and compact endosperm after MD release. However, the plumule does not immediately differentiate and elongate to push the shoot out, and this epicotyl physiological dormancy may be correlated with inhibitors in the corm [12] and the slow mobilization and transport of endospermic reserves. Seed storage matter metabolism-related genes, such as *CathB3*, *MAN2*, *MAN7*, *PROTEOLYSIS6*, and *ANNAT2,* as well as cell wall loosening genes (*EXPA4* and *EXPA8*) and polygalacturonase genes (e.g. c19523, c4968) were highly expressed in the corm and then decreased considerably during the cold stratification and seedling emergence. The β-mannanase and polygalacturonase activities in *P. sibiricum* seeds increase as the endosperm weakens and seed germination proceeds during a warm stratification [49].

## Conclusions

In summary, we analyzed seed samples at four key stratification stages to explore the molecular mechanism regulating seed germination and dormancy release. A full-length transcriptome database was established, after which the expression patterns of some dormancy-related DEGs at the Corm, Str_S, and other stages were analyzed. The results of this study have helped to further characterize the *P. sibiricum* seed dormancy trait and may form the basis of future related investigations. Specifically, we built databases comprising unigenes involved in the *P. sibiricum* seed germination process and phytohormone-related unigenes associated with seed dormancy (Table S8 and Table S11). These databases may enable researchers to further elucidate the molecular mechanism underlying seed dormancy and germination.

## Methods

### Plant materials

*Polygonatum sibiricum* Red plants are originated from Qishan County, Shanxi province, China and kindly provided by a local *P. sibiricum* growing farmer. They were identified by Dr. Jianjun Qi at the Institute of Medicinal Plant Development. These plants were then cultivated and conserved in a shaded field at the Institute of Medicinal Plant Development, Beijing, China. The seed voucher specimen (HGJG0012) was deposited at the national medicinal plant gene bank of the Institute of Medicinal Plant Development, Beijing, China. The normal field management was applied, according to our institutional field plantation guidelines. Yellow or black ripe berries were collected in early October 2016, fermented at room temperature for several days to soften the pulp outside the seeds, and then rubbed with a fine nylon mesh bag to obtain clean seeds. Fully-filled and healthy mature seeds were soaked in tap water for 1 day and then stratified in moist sand in a plastic box (10 X15 cm), which was covered with a lid to delay evaporation. Three sequential temperature stratifications were used to induce *P. sibiricum* seed germination and seedling emergence: 1) warm stratification at 25 °C for 4–6 weeks to promote radicle extrusion and cormlet formation; 2) germinated seeds with corms were subjected to a cold stratification at 4 °C for 8 weeks; 3) the cold-stratified seeds were transferred to 25 °C to induce seedling emergence. Warm and cold stratifications were conducted separately in a temperature-controlled incubator and in a refrigerator. In this study, the following *P. sibiricum* samples were collected separately: mature seeds soaked for 1 day at room temperature before the 25 °C stratification (Seed); seeds at the early germination stage during a warm stratification, with an extruded radicle (Ger-S); germinated seeds with a corm during a warm stratification (Corm); seeds at the early stage (about 4 weeks) of a cold stratification (Str), seeds at the late stage (about 8 weeks) of a cold stratification (Str_S); and seeds at the seedling emergence stage during a warm stratification (Eme). The samples were frozen with liquid nitrogen and then stored at − 80 °C for later use.

### Iso-seq library construction and PACBIO SMRT sequencing

To well represent the transcriptome information from different seed stages, we sequenced the full-length of the expressed genes in seeds of different stages (Seed, Ger-S, Corm, Str, Str_S, and Eme) using the PacBio technique. The work pipeline of our experiment to generate Iso-seq and RNA-seq data was shown in Figure S9. Total RNA was extracted from the collected *P. sibiricum* seeds (Seed, Ger-S, Corm, Str, Str_S, and Eme) using the TRIzol reagent (Invitrogen, USA) according to the manufacturer’s instructions. RNA samples with RIN values ≥7.8 were mixed equally to form an RNA pool for constructing the Iso-Seq library and the PacBio full-length cDNA sequencing. Poly-(A) RNA was isolated from the RNA pool using the oligo-(dT) magnetic bead-binding method and the Poly-(A) Purist™ Kit (Invitrogen, USA). The isolated poly-(A) RNA was eluted with RNase-free water. The mRNA (1 μg) was used as the template to synthesize cDNA with the Clontech SMARTer cDNA synthesis kit. After the PCR amplification, quality control, and purification steps were completed, the BluePippin Size Selection System was used to produce three fractions containing fragments 1–2, 2–3, and 3–6 kb long. The cDNA products were then used to construct SMRTBell Template libraries using the SMRTBell Template Prep Kit. The concentration and quality of the cDNA library were determined using the Qubit 2.0 fluorometer and the Agilent 2100 Bioanalyzer, respectively. All the operations during the library construction followed the protocols of the above-used kits. Finally, three SMRT cells were sequenced on the PacBio RS platform (Pacific Biosciences, Menlo Park, CA, USA) at Beijing Novogene Scientific Co., Ltd. (Beijing, China).

### RNA-seq library construction and sequencing

Samples collected at the Seed, Corm, Str_S, and Eme stages (three biological replicates) underwent an RNA-seq analysis. The poly-(A) mRNA was enriched from the total RNA using oligo-(dT) magnetic beads. Following the enrichment, the mRNA was fragmented into small pieces in fragmentation buffer. These fragments served as templates for the first-strand cDNA synthesis using Superscript™ III reverse transcriptase and random hexamer (N6) primers. The RNA templates were removed, after which the second cDNA strand was synthesized using dNTPs, DNA polymerase I, and RNase H. The resulting short cDNA fragments were purified with AMPure XP beads. After the end-repair and A-tailing steps, the short cDNA fragments were ligated with the Illumina paired-end adapters and purified with AMPure XP beads. Next, a PCR was used to selectively enrich DNA fragments with adapters at both ends and prepare the final cDNA library, according to the kit’s protocols. The concentrations of the cDNA libraries were determined using the Qubit 2.0 fluorometer (Life Technologies, Carlsbad, CA, USA) and their quality was evaluated using the Agilent 2100 Bioanalyzer. Finally, the 12 constructed libraries were sequenced from both ends using the Illumina HiSeq™ 2500 system (Illumina, San Diego, CA, USA) at Beijing Novogene Science Co., Ltd. (Beijing, China).

### SMRT sequencing data processing and error correction

SMRT raw sequencing data were filtered (parameters: minLength = 200 and minReadScore = 0.65) using the SMRTlink 5.1 software to produce subreads. The CCS reads were generated from subread BAM files using the following parameters: min_length 50, max_drop_fraction 0.8, no_polish TRUE, min_zscore − 9999.0, min_passes 2, min_predicted_accuracy 0.8, and max_length 15,000. The CCS reads were then classified as Flnc reads and non-full length (NFL) reads using pbclassify.py, ignorepolyA false, and minSeqLength 200. The Flnc reads were then used for the isoform-level clustering (ICE) analysis. The resulting consensus sequence of clusters (CSC) was polished in a quiver program with NFL non-chimeric reads, using the following parameters: hq_quiver_min_accuracy 0.99, bin_by_primer false, bin_size_kb 1, qv_trim_5p 100, and qv_trim_3p 30.

Additional nucleotide errors in the polished Flnc consensus sequence were corrected based on the Illumina RNA-seq data with the LoRDEC software. Redundant isoforms in the corrected consensus reads were removed with CD-HIT (−c 0.95 -T 6 -G 0 - aL 0.00 -aS 0.99) to obtain the final non-redundant reference transcripts for the subsequent analyses.

### Gene functional annotation

Non-redundant Flnc transcripts were annotated based on BLAST searches of the following seven databases: Nr (NCBI non-redundant protein sequences), Nt (NCBI non-redundant nucleotide sequences), Pfam (Protein family), KOG/COG (Clusters of Orthologous Groups of proteins), Swiss-Prot (a manually annotated and reviewed protein sequence database), KEGG (Kyoto Encyclopedia of Genes and Genomes), and GO (Gene Ontology). The programs used for the functional annotation included hmmscan (version: 3.1b2) for the Pfam database analysis, blast+ (version: 2.6.0+) for the Nt database analysis, and diamond blastx (version: 0.8.36) for the Nr, KOG/COG, Swiss-Prot, KEGG, and GO database analyses. The E-cutoff value for all seven database analyses was set as ≤1e-5. The top hit for the BLAST results was used for the functional annotation. The open reading frame of each FL transcript was predicted using the ANGLE pipeline.

Hormone metabolism and signaling genes were annotated based on AraCyc (v17.1) and the KEGG database as well as the relevant published literature regarding Arabidopsis. Transcription factors and regulators were identified and classified using the iTAK program (version 1.7a) (https://github.com/kentnf/iTAK) and the associated database.

### Gene expression levels and differential expression analysis

The Illumina RNA-seq clean reads for 12 samples were separately mapped onto the above-mentioned Flnc transcript sequences using the bowtie2 program in the RSEM software. The mapped read count of each transcript was calculated and further transformed into FPKM values, which were used as the expression levels in different samples. The DEGs between two compared stages were determined using the DESeq R package (1.10.1), with the following criteria: |log_2_ (fold-change)| ≥ 1 and adjusted *p* value < 0.05.

The GO and KEGG enrichment analyses of the DEGs were performed using the GOseq R package (version 1.10.1) and the KOBAS software (version 2.0), respectively. The significance of the enriched GO terms and enriched KEGG pathways was separately assessed with the Wallenius non-central hyper-geometric distribution test and the hyper-geometric distribution test. An adjusted *p* value < 0.05 was set as the threshold for significance.

### qRT-PCR validation of differentially expressed genes

To verify the transcriptome sequencing results, the expression levels of 15 unigenes involved in ABA/GA metabolism and signaling pathways in *P. sibiricum* seeds collected at the Seed, Corm, Str_S, and Eme stages were analyzed by qRT-PCR. Total RNA was extracted using the EASYspin Plus Complex Plant RNA kit (Aidlab, Beijing, China) and reverse transcribed to generate first-strand cDNA using the TUREscript 1st Strand cDNA Synthesis kit (Aidlab). The cDNA was used for the qRT-PCR analysis involving gene-specific primers (Table S12) and 2× SYBR Green qPCR Master Mix (Aidlab). The qRT-PCR was performed using the CFX96™ Realtime PCR System (Bio-Rad, USA), with the following program: 94 °C for 3 min; 40 cycles of 94 °C for 15 s, 55 °C for 15 s, and 72 °C for 20 s; and a melting curve analysis from 65 to 95 °C for 5 s. The 2^−ΔΔCt^ method was used to calculate the expression levels of the analyzed genes [50]. A *GAPDH* gene that showed the most stablest expression among our four seed samples was used as the internal reference for normalizing and comparing the expression levels of the analyzed genes among different samples.

## Supplementary Information

**Additional file 1: **The online version contains supplementary material available at XXX. **Figure S1**. Length distribution of the predicted coding sequences in *P. sibiricum* transcriptome. **Figure S2.** KOG annotation of *P. sibiricum* unigenes. **Figure S3.** GO annotation of *P. sibiricum* unigenes. **Figure S4.** FPKM distribution of *P. sibiricum* unigenes in different samples. **Figure S5**. PCA (**A**) and Pearson correlation analysis (**B**) of *P. sibiricum* samples. **Figure S6.** Expression patterns of differentially-expressed hormone metabolic and signaling genes during seed dormancy breaking and seedling establishment. **Figure S9**. The pipeline of studying molecular regulation of *P. sibiricum* seed dormancy and germination based on PacBio SMRT-Seq and Illumination RNA-Seq. **Table S1.** *P. sibiricum* unigenes containing different number of transcripts. **Table S2.** Statistics of functional annotation of *P. sibiricum* transcriptome. **Table S7.** RNA-seq mapped reads. **Table S10.** Total number/DEG number of each TF and TR family.

**Additional file 2: Table S3.** Homologous species distribution of *P. sibiricum* unigenes annotated in the Nr database.

**Additional file 3: Table S4.***P. sibiricum* unigenes aligned to Polynatum genes in Nr database.

**Additional file 4: Table S5.** GO annotation of *P. sibiricum* unigenes.

**Additional file 5: Table S6.** KEGG pathway annotation of *P. sibiricum* unigenes.

**Additional file 6: Table S8.** List of hormone metabolic and signaling genes in *P. sibiricum* transcriptome.

**Additional file 7: Figure S7.** qRT-PCR and RNA-seq results of representative DEGs involved in ABA metabolism and signaling pathway.

**Additional file 8: Figure S8**. qRT-PCR and RNA-seq results of representative DEGs involved in GA metabolism and signaling pathway.

**Additional file 9: Table S9.** Transcription factors and regulators in *P. sibiricum* transcriptome.

**Additional file 10: Table S11.** List of *P. sibiricum* genes homologous to Arabidopsis known seed dormancy and germination genes.

**Additional file 11: Table S12.** List of *P. sibiricum* genes for RT-qPCR validation.

## Data Availability

The raw sequence data have been deposited in the Genome Sequence Archive of the BIG Data Center (Beijing Institute of Genomics, Chinese Academy of Sciences, https://ngdc.cncb.ac.cn/) (Accession No. CRA003484). All data generated or analyzed during this study are available from the corresponding author on reasonable request.

## Abbreviations

*P. sibiricum *: *Polygonatum sibiricum*
MPD: Morphophysiological dormancy
SMRT: Single-molecule real-time
DEGs: Differentially Expressed Genes
qRT-PCR: Quantitative real-time polymerase chain reaction
TF: Transcription factor
Iso-seq: Isoform sequencing
PacBio: Pacific Biosciences
CCS: Circular consensus sequence
Flnc: Full-length non-chimeric
FPKM: Fragments per kilobase of exon model per million mapped reads
ZEP: Zeaxanthin epoxidase
AAO3: Abscisic-aldehyde oxidase
CYP707A: Abscisic acid 8′-hydroxylase
ABI5: Abscisic acid insensitive protein 5
ABF: ABA-responsive element-binding factor
PYL4/8: Abscisic acid receptor
PP2C: Protein phosphatase 2C
GA3ox: Gibberellin 3-oxidase
GAMT2: Gibberellin A4 carboxyl methyltransferase 2
GID1: Gibberellin-insensitive dwarf 1
GASA3/6/14: Gibberellin-regulation protein
CIGR2: Chitin-inducible gibberellin-responsive protein 2
