# Supplementary material for: Transcriptome profiles revealed molecular mechanisms of alternating temperatures in breaking the epicotyl morphophysiological dormancy of Polygonatum sibiricum seeds
Source: BMC Plant Biol. 2021 Aug 12;21:370. doi: 10.1186/s12870-021-03147-7 (PMC8359049; doi:10.1186/s12870-021-03147-7)
Supplement: Supplementary file 1 — Additional file 1: The online version contains supplementary material available at XXX. Figure S1. Length distribution of the predicted coding sequences in P. sibiricum transcriptome. Figure S2. KOG annotation of P. sibiricum unigenes. Figure S3. GO annotation of P. sibiricum unigenes. Figure S4. FPKM distribution of P. sibiricum unigenes in different samples. Figure S5. PCA (A) and Pearson correlation analysis (B) of P. sibiricum samples. Figure S6. Expression patterns of differentially-expressed hormone metabolic and signaling genes during seed dormancy breaking and seedling establishment. Figure S9. The pipeline of studying molecular regulation of P. sibiricum seed dormancy and germination based on PacBio SMRT-Seq and Illumination RNA-Seq. Table S1. P. sibiricum unigenes containing different number of transcripts. Table S2. Statistics of functional annotation of P. sibiricum transcriptome. Table S7. RNA-seq mapped reads. Table S10. Total number/DEG number of each TF and TR family. [file 12870_2021_3147_MOESM1_ESM.docx]

**
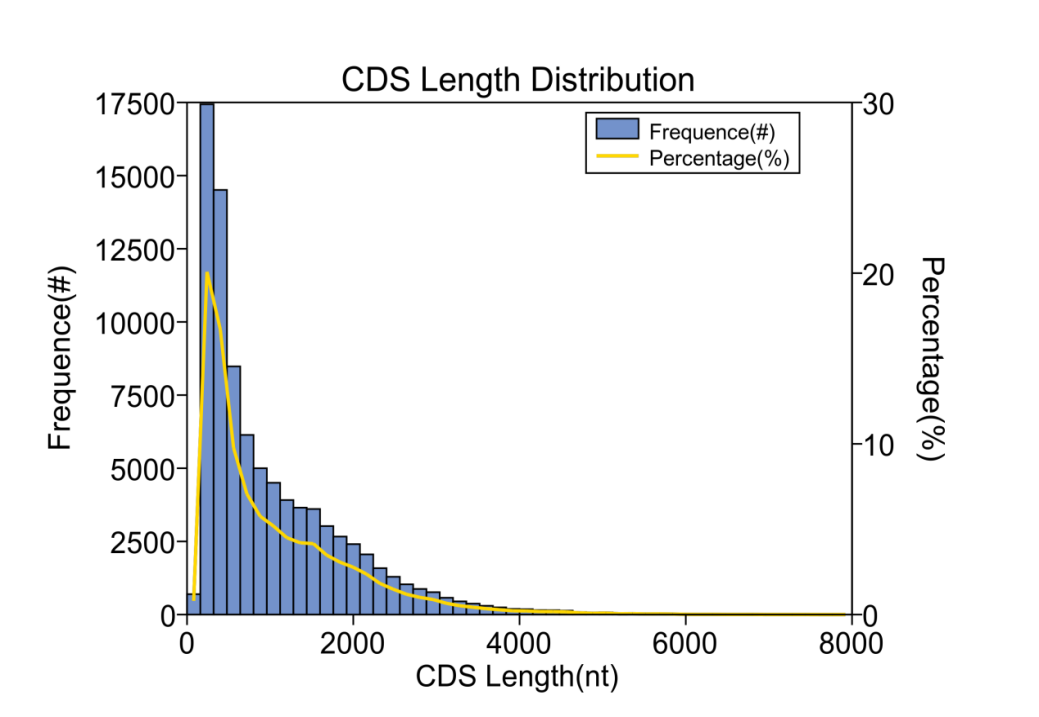
**

**
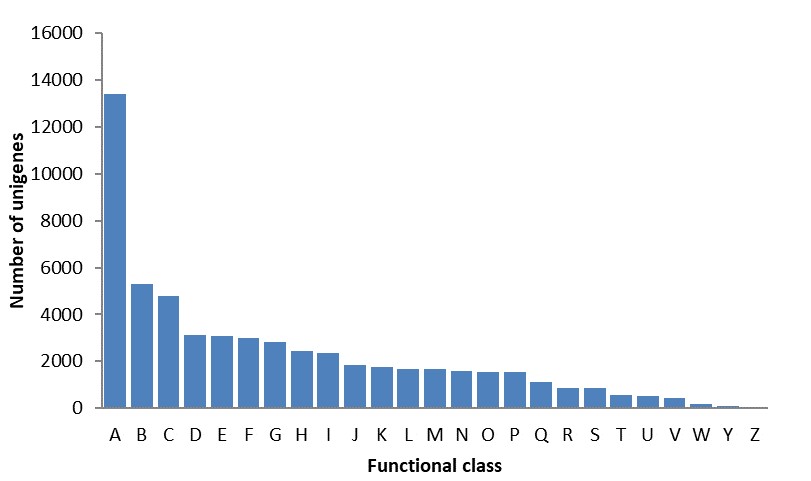
Figure S1.** Length distribution of the predicted coding sequences in *P. sibiricum* transcriptome.

**Figure S2.** KOG annotation of *P. sibiricum* unigenes. The x-axis represents 25 KOG categories (**A**-**Z**), the y-axis represents the number of annotated unigenes. **A.** General function prediction only; **B**. Posttranslational modification, protein turnover, chaperones; **C.** Signal transduction mechanisms; **D.** RNA processing and modification; **E.** Transcription; **F.** Intracellular trafficking, secretion, and vesicular transport; **G.** Translation, ribosomal structure and biogenesis; **H.** Carbohydrate transport and metabolism; **I.** Function unknown; **J.** Lipid transport and metabolism; **K.** Amino acid transport and metabolism; **L.** Secondary metabolites biosynthesis, transport and catabolism; **M.** Energy production and conversion; **N.** Replication, recombination and repair; **O.** Inorganic ion transport and metabolism; **P.** Cell cycle control, cell division, chromosome partitioning; **Q.** Cytoskeleton; **R.** Chromatin structure and dynamics; **S.** Cell wall/membrane/envelope biogenesis; **T.** Nucleotide transport and metabolism; **U.** Coenzyme transport and metabolism; **V.** Nuclear structure; **W.** Defense mechanisms; **Y.** Extracellular structures; **Z.** Cell motility.


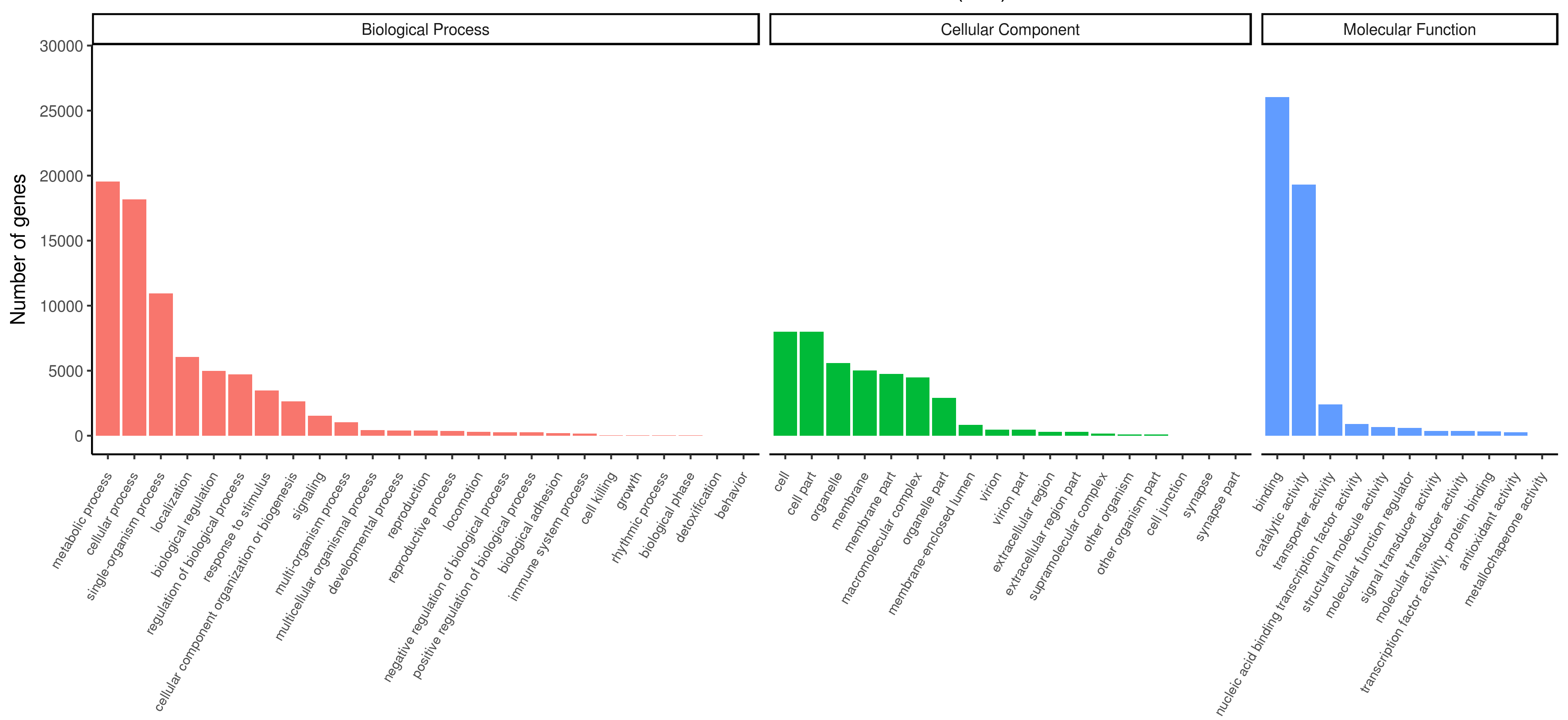
**Figure S3.** GO annotation of *P. sibiricum* unigenes. The x-axis represents 2^nd^ GO terms at three main categories (biological process, cellular component, molecular function), the y-axis represents the number of annotated unigenes in each GO term (level 2).

**
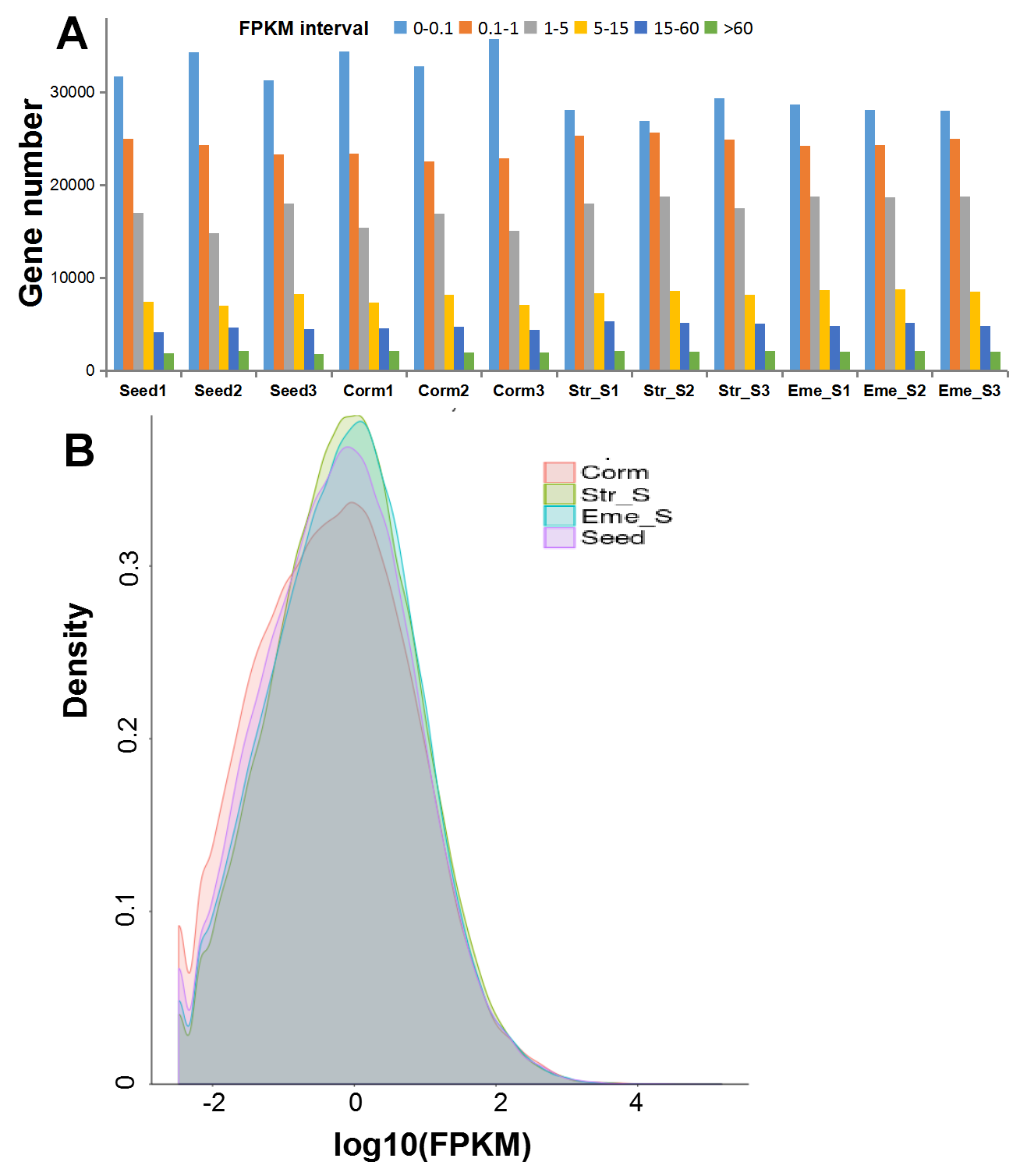
**

**Figure S4.** FPKM distribution of *P. sibiricum* unigenes in different samples. **A.** FPKM interval distribution; **B.** FKPM density distribution.



**Figure S5**. PCA (**A**) and Pearson correlation analysis (**B**) of *P. sibiricum* samples.

**

Figure S6.** Expression patterns of differentially-expressed hormone metabolic and signaling genes during seed dormancy breaking and seedling establishment. (**A**) Hierarchical clustering. Red and blue represented increased and decreased transcript abundance, respectively. Main subcluster No. 1-10 were marked on left branching points. (**B**) Number of DEGs in Cluster 1-10. three numbers under each hormone referred to number of DEGs involved in biosynthesis, degradation/inactivation and signaling transduction.


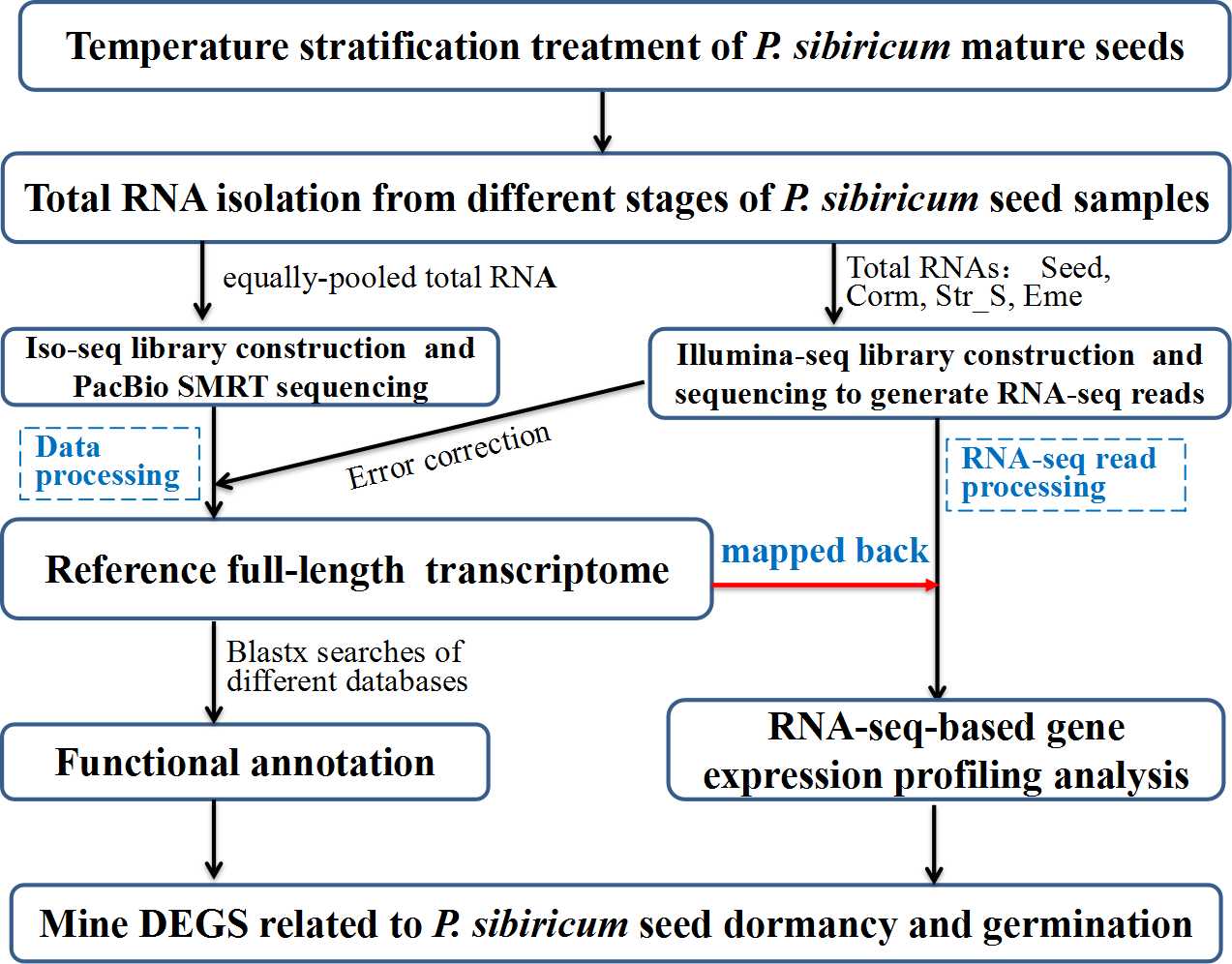


**Figure S9**. The pipeline of studying molecular regulation of *P. sibiricum* seed dormancy and germination based on PacBio SMRT-Seq and Illumination RNA-Seq.

**Table S1.** *P. sibiricum* unigenes containing different number of transcripts.

| Transcript number | 1 | 2 | 3 | 4 | 5 | 6 | 7 | 8 | 9 | >10 |
| --- | --- | --- | --- | --- | --- | --- | --- | --- | --- | --- |
| Unigene number | 71936 | 8194 | 2859 | 1396 | 827 | 539 | 330 | 270 | 163 | 737 |
| % of the total | 82.45 | 9.39 | 3.28 | 1.60 | 0.95 | 0.62 | 0.38 | 0.31 | 0.19 | 0.84 |

**Table S2.** Statistics of functional annotation of *P. sibiricum* transcriptome.

| **Annotated database** | **annotated number (%)** | **300 bp≤length<1000 bp** | **length≥1000 bp** |
| --- | --- | --- | --- |
| Nr | 74921 (85.87%) | 3962(5.29%) | 70890(94.62%) |
| Nt | 54583 (62.56%) | 2484(4.55%) | 52043(95.34%) |
| Swiss-port | 61678 (70.69%) | 2948(4.78%) | 58693(95.16%) |
| KOG | 50906 (58.34%) | 1945(3.82%) | 48942(96.14%) |
| KEGG | 73525 (84.27%) | 3676(5.00%) | 69791(94.92%) |
| GO | 41807 (47.92%) | 1834(4.39%) | 39943(95.54%) |
| Pfam | 41807 (47.92%) | 1155(2.76%) | 39943(95.54%) |
| at least one database | 77148 (88.42%) | 4320(5.60%) | 72709(94.25%) |
| All databases | 25521 (29.25%) | 788(3.09%) | 24732(96.91%) |

**Table S7.** RNA-seq mapped reads.

| **Sample** | **Total Clean Reads** | **Number of Mapped reads** | **Mapped reads (%)** |
| --- | --- | --- | --- |
| Seed1 | 53,793,192 | 41,064,324 | 76.34% |
| Seed2 | 47,285,766 | 25,520,382 | 53.97% |
| Seed3 | 81,421,838 | 62,059,012 | 76.22% |
| Corm1 | 56,240,236 | 39,726,076 | 70.64% |
| Corm2 | 102,039,170 | 76,830,910 | 75.30% |
| Corm3 | 53,781,168 | 40,704,906 | 75.69% |
| Str_S1 | 52,514,256 | 35,777,726 | 68.13% |
| Str_S2 | 56,671,922 | 39,614,020 | 69.90% |
| Str_S3 | 46,833,512 | 32,572,494 | 69.55% |
| Eme-S1 | 51,054,124 | 35,554,500 | 69.64% |
| Eme-S2 | 51,267,252 | 36,139,868 | 70.49% |
| Eme-S3 | 64,086,482 | 45,314,634 | 70.71% |

**Table S10.** Total number/DEG number of each TF and TR family.

1. **TF**

| **FAR1**: 236/90 | **C3H** : 214/68 | **C2H2** : 197/56 | **MYB-related** :162/67 | **WRKY :**148/67 | **bHLH**: 138/63 |
| --- | --- | --- | --- | --- | --- |
| **B3-AR**F:116/52 | **NAC**: 105/51 | **bZIP**: 94/34 | **GRAS**: 80/40 | **SBP**: 77/24 | **GARP-G2-lik**e: 70/23 |
| **B3**: 63/27 | **MYB**:59/32 | **TUB**: 57/19 | **AP2/ERF-ERF**:53/28 | **HB-HD-ZIP**: 52/16 | **Trihelix**: 49/19 |
| **HB-other**: 48/17 | **C2C2-GATA**: 45/23 | **HSF**: 45/16 | **HB-BELL**: 44/21 | **zn-clus**: 35/1 | **BES1**:31/12 |
| **GARP-ARR-B**: 28/8 | **EIL**: 24/11 | **RWP-RK**: 23/8 | **NF-YA**: 21/6 | **TCP**: 21/11 | **CAMTA**: 20/7 |
| **LOB**: 20/9 | **C2C2-Dof**: 16/11 | **AP2/ERF-AP2** :15/6 | **C2C2-LSD**: 15/4 | **Tify**: 15/7 | **C2C2-CO-like**:14/6 |
| **CPP**: 14/9 | **PLATZ**: 13/7 | **NF-X1**: 12/3 | **NF-YC**: 12/6 | **E2F-DP**: 11/5 | **GRF: 10/7** |
| **Alfin-like**: 9/2 | **BBR-BPC**: 9/1 | **CSD**: 5/1 | **HB-PHD**: 5/2 | **MADS-M-type**: 5/0 | **S1Fa-like**: 5/1 |
| **zf-HD**: 5/2 | **C2C2-YABBY**: 4/1 | **GeBP**: 4/0 | **LIM**: 4/0 | **SRS**: 4/3 | **Whirly**: 4/1 |
| **DBB**: 3/1 | **ULT**: 3/1 | **HB-KNOX**: 2/0 | **NF-YB**: 2/2 | **OFP**: 2/1 | **AP2/ERF-RAV**: 1/1 |
| **DBP**: 1/0 | **HB-WOX**: 1/1 | **HRT**: 1/0 | **MADS-MIKC**: 1/0 | **SAP**: 1/0 | **STAT**:1/0 |
| **VOZ**: 1/0 |  |  |  |  |  |

1. **TR**

| **SNF2**: 280/108 | **Others**: 209/68 | **PHD**: 153/58 | **SET**: 152/60 | **Jumonji**: 102/37 | **IWS1**: 94/17 |
| --- | --- | --- | --- | --- | --- |
| **SOH1**: 70/11 | **TRAF**: 65/23 | **AUX/IAA**: 62/22 | **TAZ**: 61/21 | **SWI/SNF-BAF60b**: 48/12 | **ARID**: 47/20 |
| **DDT**: 34/15 | **LUG**: 34/10 | **mTERF**: 33/7 | **GNAT**: 24/6 | **RB**: 22/10 | **HMG**: 18/5 |
| **SWI/SNF-SWI3**:  14/4 | **Pseudo ARR-B**: 12/4 | **Coactivator p15**: 7/1 | **Rcd1-like**: 4/0 | **MBF1**: 3/0 | **MED6**: 3/0 |
| **MED7**: 1/0 |  |  |  |  |  |
