# Supplementary figures and images for: Transcriptome profiles revealed molecular mechanisms of alternating temperatures in breaking the epicotyl morphophysiological dormancy of Polygonatum sibiricum seeds
Source: BMC Plant Biol. 2021 Aug 12;21:370. doi: 10.1186/s12870-021-03147-7 (PMC8359049; doi:10.1186/s12870-021-03147-7)

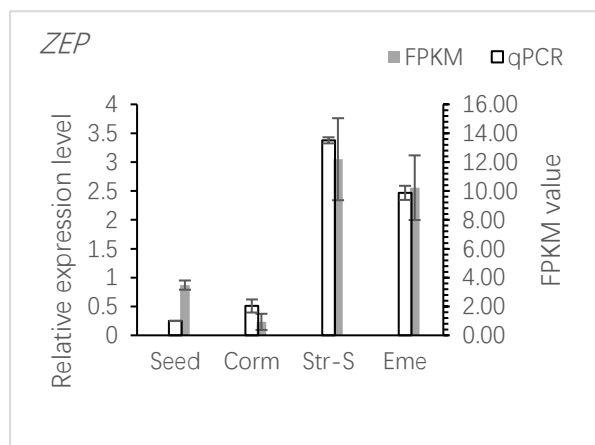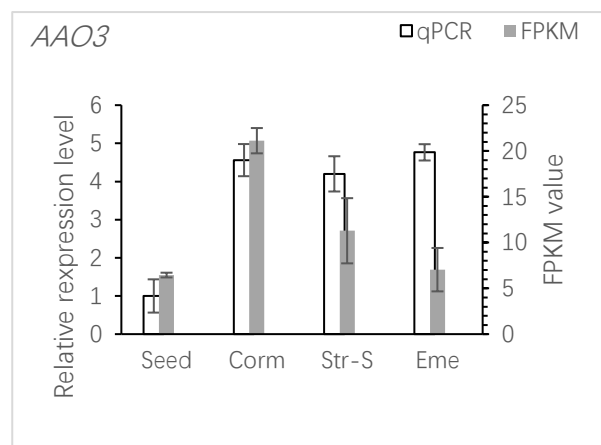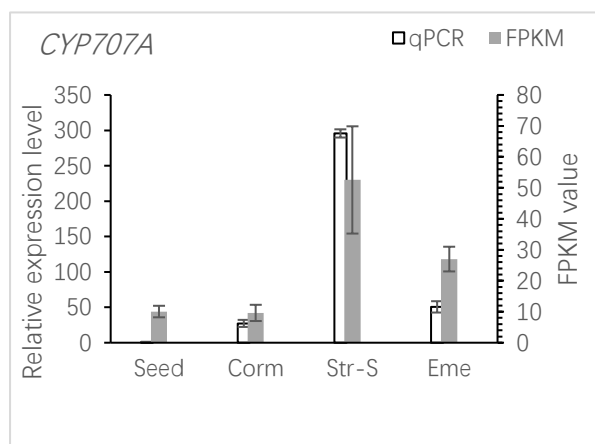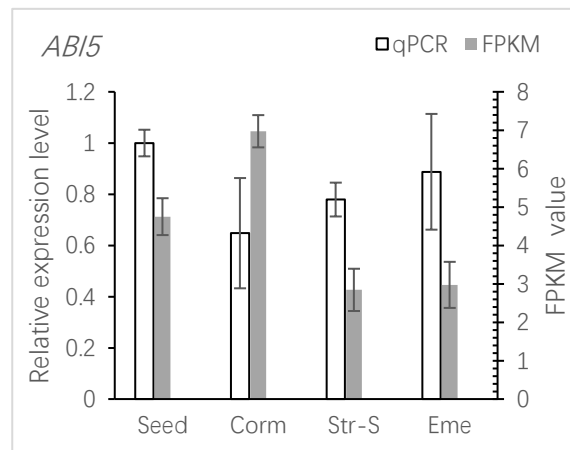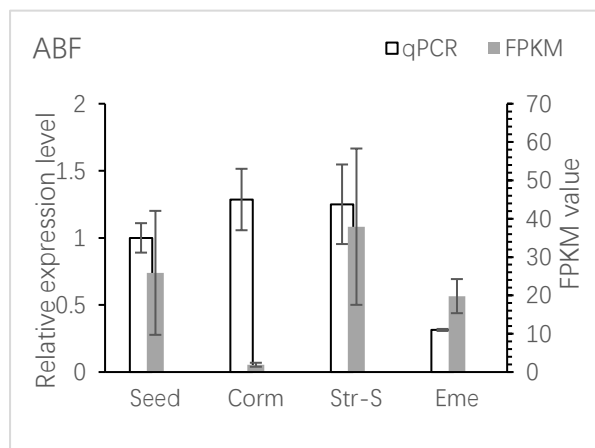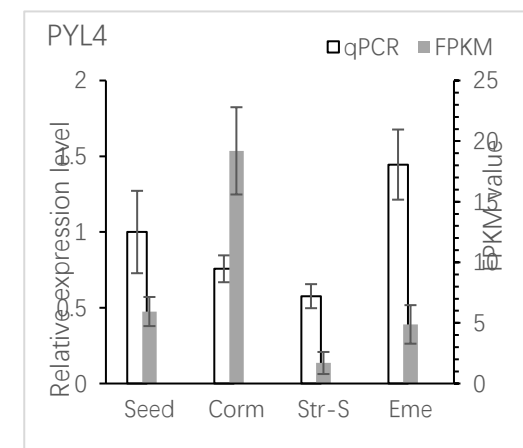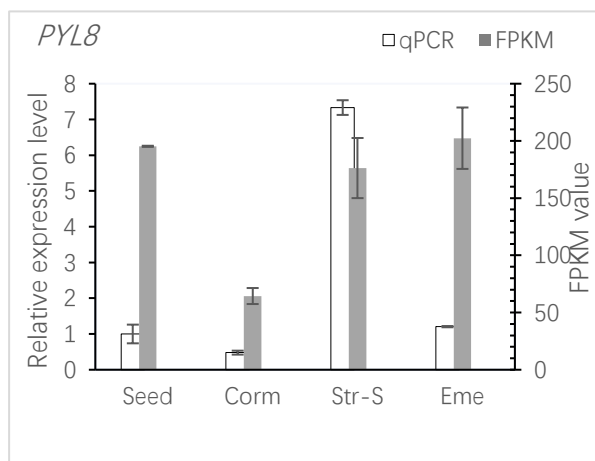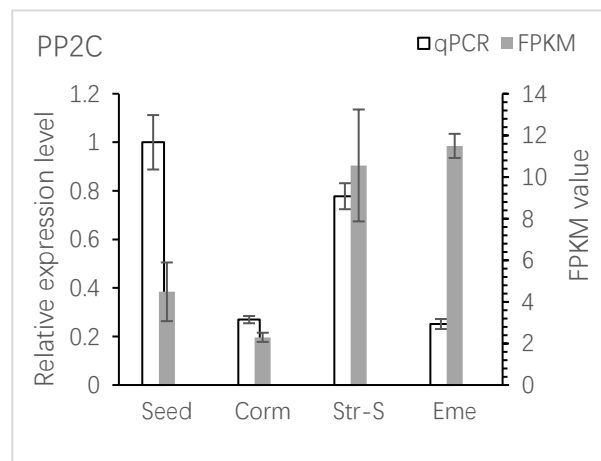

Supplement: Supplementary file 7 — Additional file 7: Figure S7. qRT-PCR and RNA-seq results of representative DEGs involved in ABA metabolism and signaling pathway. [file 12870_2021_3147_MOESM7_ESM.pdf]

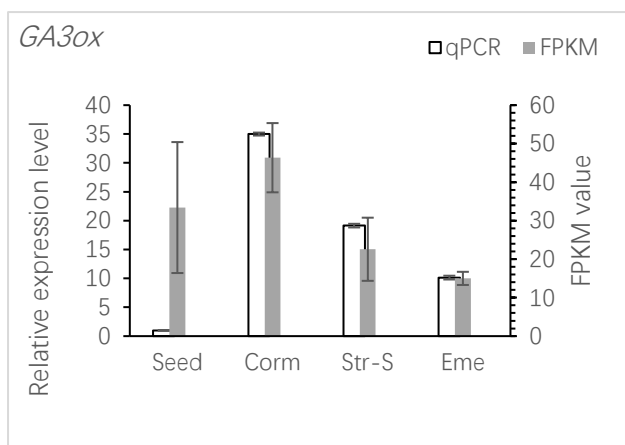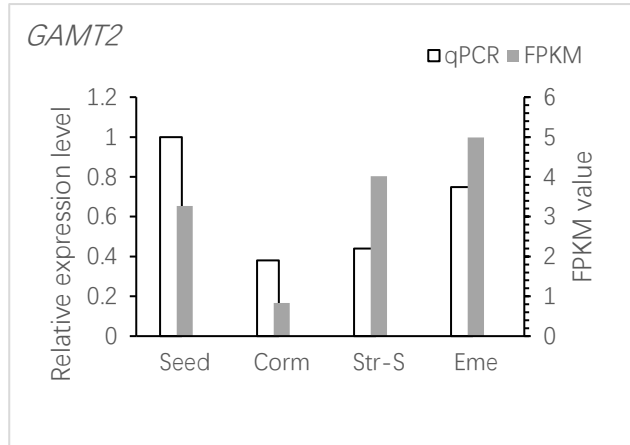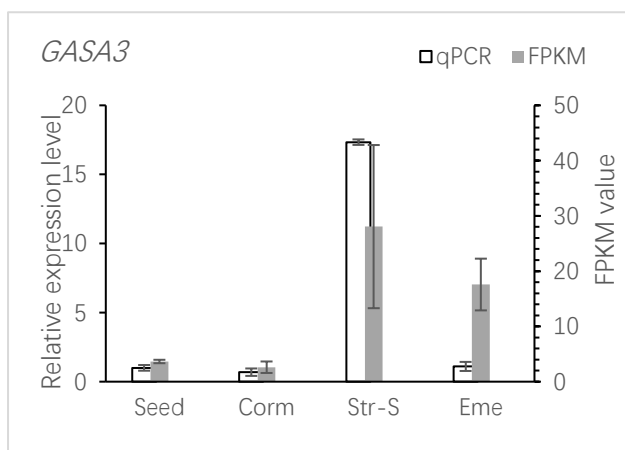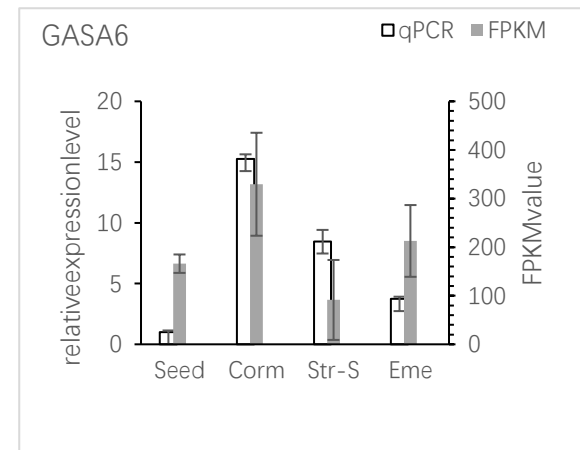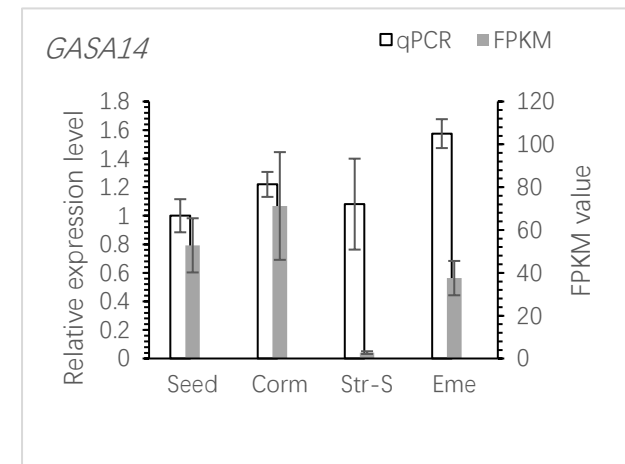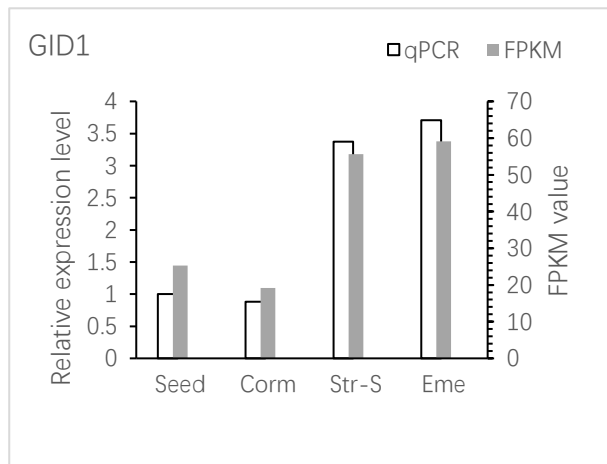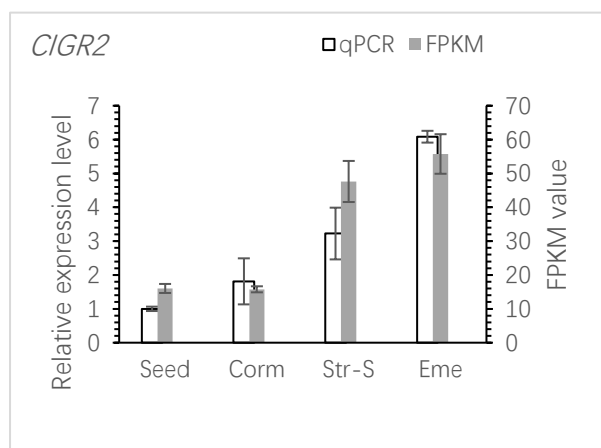

Supplement: Supplementary file 8 — Additional file 8: Figure S8. qRT-PCR and RNA-seq results of representative DEGs involved in GA metabolism and signaling pathway. [file 12870_2021_3147_MOESM8_ESM.pdf]
